# Musashi 2 influences chronic lymphocytic leukemia cell survival and growth making it a potential therapeutic target

**DOI:** 10.1038/s41375-020-01115-y

**Published:** 2021-01-27

**Authors:** Florencia Palacios, Xiao-Jie Yan, Gerardo Ferrer, Shih-Shih Chen, Stefano Vergani, Xuejing Yang, Jeffrey Gardner, Jaqueline C. Barrientos, Philip Rock, Richard Burack, Jonathan E. Kolitz, Steven L. Allen, Michael G. Kharas, Omar Abdel-Wahab, Kanti R. Rai, Nicholas Chiorazzi

**Affiliations:** 1 Karches Center for Oncology Research, The Feinstein Institutes for Medical Research, Northwell Health, Manhasset, NY USA; 2 Molecular Pharmacology Program, Center for Cell Engineering, Center for Stem Cell Biology, Center for Experimental Therapeutics, Memorial Sloan Kettering Cancer Center, New York, NY USA; 3 Human Oncology and Pathogenesis Program, Memorial Sloan Kettering Cancer Center, New York, NY USA; 4 Department of Medicine, Northwell Health, Manhasset and New Hyde Park, New York, NY USA; 5 Department of Medicine, Donald and Barbara Zucker School of Medicine at Hofstra/Northwell, Hempstead, NY USA; 6 Department of Pathology, University of Rochester, Rochester, NY USA

**Keywords:** Translational research, Chronic lymphocytic leukaemia

## Abstract

Progression of chronic lymphocytic leukemia (CLL) results from the expansion of a small fraction of proliferating leukemic B cells. When comparing the global gene expression of recently divided CLL cells with that of previously divided cells, we found higher levels of genes involved in regulating gene expression. One of these was the oncogene Musashi 2 (MSI2), an RNA-binding protein that induces or represses translation. While there is an established role for MSI2 in normal and malignant stem cells, much less is known about its expression and role in CLL. Here we report for the first time ex vivo and in vitro experiments that MSI2 protein levels are higher in dividing and recently divided leukemic cells and that downregulating MSI2 expression or blocking its function eliminates primary human and murine CLL and mature myeloid cells. Notably, mature T cells and hematopoietic stem and progenitor cells are not affected. We also confirm that higher MSI2 levels correlate with poor outcome markers, shorter time-to-first-treatment, and overall survival. Thus, our data highlight an important role for MSI2 in CLL-cell survival and proliferation and associate MSI2 with poor prognosis in CLL patients. Collectively, these findings pinpoint MSI2 as a potentially valuable therapeutic target in CLL.

## Introduction

Chronic lymphocytic leukemia (CLL) is a common, incurable adult hematologic disease of unknown etiology [1–3]. Although the majority of circulating CLL cells are not replicating, a small fraction divides in lymphoid tissues and some of the recently divided leukemic cells migrate and are found in peripheral blood (PB) [4]. Importantly, the rate of growth of CLL clones correlates directly with poor outcome measured as shorter time-to-first-treatment [5]. Furthermore, since dividing cells upregulate DNA mutators, such as AID [6–8], cells within this intraclonal subset can acquire new DNA abnormalities that could lead to more lethal disease. This makes cells of this intraclonal fraction important targets for therapy.

When performing gene expression profiling (GEP), we found that the RNA-binding protein Musashi 2 (MSI2) was highly expressed in dividing or recently divided cells (proliferative fraction, PF), defined by the CXCR4^Dim^CD5^Bright^ immunophenotype compared with the resting fraction (RF, CXCR4^Bright^CD5^Dim^). *MSI* was initially discovered in Drosophila, in which it mediates asymmetric cell division during bristle development [9]. Moreover, MSI2 regulates self-renewal and differentiation of neuronal [9] and hematopoietic stem cells (HSCs) [10–12] by modulating protein translation. MSI2 regulates expression of proteins post-transcriptionally by binding to target mRNAs via two RNA recognition motifs [13–15] either positively [16–18] by interacting with poly(A) binding protein [19] or negatively by blocking protein translation [14, 15].

Remarkably, excessive MSI2 expression associates with tumorigenesis and poor prognosis in multiple cancers [17, 20–25] as well as in different types of leukemias [10, 26–30]. In CLL, although high MSI2 levels can correlate with poor outcome [27, 31, 32], there are no studies addressing the function of MSI2 in CLL, how this affects leukemia-cell survival and growth and how this influences clinical outcome in patients or animal models. Therefore, we have studied the biological role of MSI2 in CLL cells, and its association with leukemic B-cell proliferation and survival and in patient outcome.

## Materials and methods

### Patients

The study was approved by the Institutional Review Boards of Northwell Health and Memorial Sloan Kettering Cancer Center (MSKCC) and was conducted according to the principles of the World Medical Association Declaration of Helsinki. CLL patients were diagnosed as recommended [33], and all subjects provided written informed consent at enrollment. Procedures for the isolation of malignant cells and the determination of their purity have been described previously [34]. Normal B lymphocytes were collected from the PB of healthy age-matched individuals and provided by the New York Blood Center.

### Measurements of surface and intracellular antigens by flow cytometry

PBMCs were incubated with different combinations of murine anti-human mAbs directly conjugated with the indicated fluorochrome (Table S2). For further information, see Supplementary Methods.

### Gene expression profiling (GEP) and gene expression data analyses of isolated CLL intraclonal fractions

CLL cell fractions were isolated on the basis of expression of CXCR4 and CD5 [35]. RNA extraction and GEP were performed as described [35].

### Culture conditions for B-cell stimulation

PBMC or B cells from CLL patients or HDs were cultured in an enriched medium [36] as described in Supplementary Methods.

### In vitro kinase inhibition

CLL PBMCs were cultured with CpG-ODN + IL15 for 4 days. The day before harvesting the cells, 30, 40, and 50 μM of Pi3K inhibitor (LY294002, Cell Signaling) or 20, 200, and 2000 nM of ERK inhibitor (SCH772984, MedChem Express) were added. When using the Btk inhibitor, ibrutinib, CLL PBMCs were cultured with CpG-ODN + IL15 for 4 days, and then 0.5, 1, and 2 μM of BTKi were added and the cells cultured for 72 h. Live CD19^+^CD5^+^ B cells were evaluated for MSI2 levels as well as cells at different phases of the cell cycle by flow cytometry, as described in Supplementary Methods.

### MSI2 siRNA knockdown

Accell SMARTpool siRNA targeting human MSI2 (siMSI2) and a non-targeting negative pool control siRNA (siCTR) were used in Accell siRNA delivery medium (Horizon). MSI2 expression was reduced by culturing 1 × 10^5^ cells/mL with siRNAs (1 μM) for 4 days. In certain experiments, siRNAs (1 μM) plus CLL cells (2 × 10^6^ cells/ml) were cultured with HS5 cells [37] (50:1), or CpG-ODN + IL15, or both for 4 days, and MSI2 mRNA levels were determined by semi-quantitative or real-time qPCR and protein levels measured by flow cytometry. Primers used to amplify MSI2 (266 base pairs) and βactin (250 base pairs) were: MSI2 Forward 3′ ATGGGAGCCAAGGCACCTC 5′, MSI2 reverse 3′ TCAATCGTCTTGGAATCTAACTC 5′: β-actin 3′ GAGCGCGGCTACAGCTTCAC 5′, β-actin reverse 3′ GTGTAACGCAACTAAGTCAT 5′.

The absolute number and percentage of viable cells was determined using counting bright beads (CountBright™ Absolute Counting Beads, Thermo Fisher Scientific) and Annexin V-PE (BD Bioscience) plus 4′,6-diamidino-2-phenylindole (DAPI), respectively.

### Human cell apoptosis array

MEC1 cells were treated for 4 days with siRNAs, protein extracts were prepared, and analyses conducted as per the manufacturer’s instructions (Proteome Profiler Human Apoptosis Array Kit, R&D). The intensity score of duplicate array spots was measured with ImageJ software program, and the averaged intensity was calculated by subtracting the averaged background signal. Fold change was obtained by comparing the data of siMSI2 and siCTR treatments.

### In vitro treatment of primary cells with MSI2 inhibitor

CLL PBMCs or B cells were cultured as above without or with different concentrations (5, 10, and 20 μM) of the MSI2 inhibitor, Ro 08-2750, for 1, 2, and 3 days. Live cells were surface stained with antihuman CD19, CD5 and CXCR4 mAbs and the absolute number and percentage of viable cells determined using counting bright beads and Annexin V-PE plus DAPI, respectively.

### Mice

CB17 SCID mice were housed under conventional barrier protection and procedures were performed in accordance with The Feinstein Institute’s Institutional Animal Care and Use Committee requirements.

### Statistics

Groups of patients were compared using the Mann–Whitney *U*-test. Spearman’s rank test was used to evaluate correlations. The Maximally Selected Rank Statistics (maxstat) package for R-2.8.0 was used to optimize cut-off points for the studied variables [19]. Time-to-first-treatment (TTFT) and overall survival (OS) were calculated from the sample date to the date of initial therapy or sample date to the day of last follow-up. Curves were calculated by the Kaplan–Meier method; comparisons between groups were performed by the log-rank test.

Additional Materials and Methods are described in Supplementary Methods.

## Results

### CLL cells contain higher MSI2 protein levels than healthy donor B lymphocytes, with the highest levels found in the proliferative fraction

**Fig. 1:**
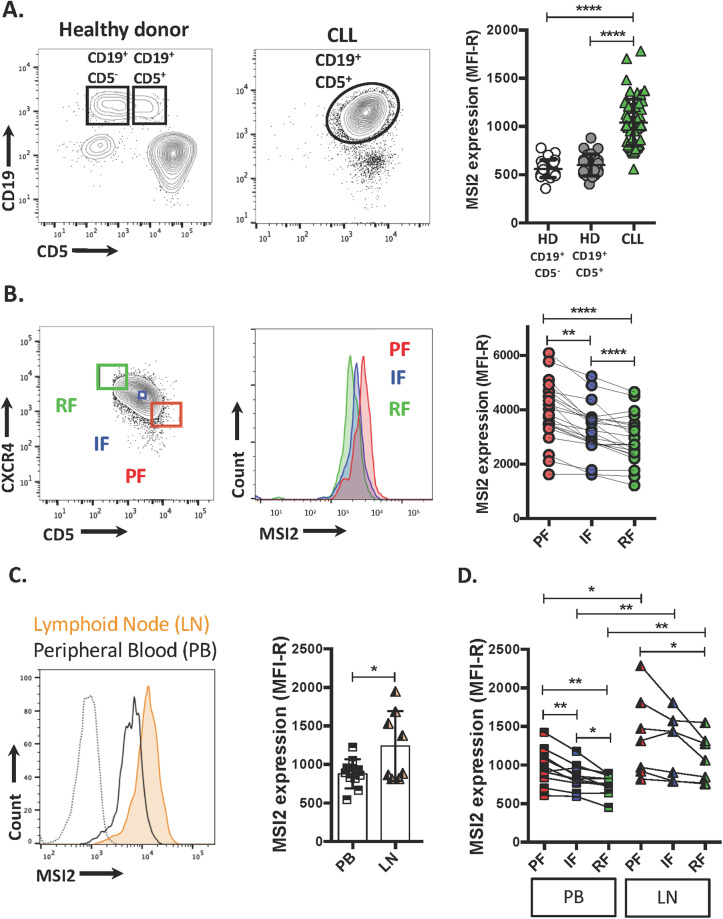
MSI2 levels are high in CLL cells and even higher in the proliferative fraction of CLL clones. **A** Representative flow cytometry profile of CD19 and CD5 surface expression from a HD and a CLL patient (left, middle panel). MSI2 expression (relative mean fluorescence intensity, MFI-R) in CLL B cells (CD19^+^CD5^+^, green triangles) compared to HD B cells (CD19^+^CD5^+^ or CD19^+^CD5^−^, gray and white circles) (right). **B** Representative flow cytometry profile of CXCR4/CD5 surface expression on a CLL clone showing the PF (CXCR4^Dim^CD5^Bright^), IF (CXCR4^Int^CD5^Int^), and RF (CXCR4^Bright^CD5^Dim^) on the left. Representative flow cytometry histogram of MSI2 expression in PF (red), IF (blue), and RF (green) (middle panel). Relative MSI2 expression in each CXCR4/CD5 fraction in CLL patients from 22 cases (right). **C** Representative flow cytometry histogram of MSI2 expression in CD19^+^CD5^+^ cells from PB (black line) and LN (orange) (left). The isotype control is shown as a dashed line. MFI-R in CD19^+^CD5^+^ from CLL samples from the different tissues (PB, *n* = 10 and LN *n* = 8). **D** MFI-R in each CXCR4/CD5 fraction. Statistical analysis was performed using One-way ANOVA, Tukey’s multiple comparison test. Shown are individual values. *P < 0.05; ** *P* < 0.01; ***P < 0.001; ***P < 0.0001.

GEP indicated that MSI2 mRNA levels are higher in B cells from 26 CLL patients than 11 healthy donors (HD) (Fig. S1A). To verify these results, we simultaneously studied MSI2 levels in 55 CLL patients (Table S1A) and 25 HDs by flow cytometry, documenting that CLL cells display higher levels of MSI2 protein than HD B cells of both the CD5^+^ and CD5^−^ subsets (Fig. 1A).

In addition, since our mRNA data indicated that within CLL clones the PF exhibits higher levels of MSI2 than the RF (Fig. S1B), we evaluated MSI2 protein amounts in these intraclonal fractions that differ in time since last cell division. The PF exhibited higher MSI2 levels than the IF, and the IF higher than the RF (MSI2-PF > MSI2-IF > MSI2-RF) (Fig. 1B). Comparing the ratios of MSI2 levels between the three CXCR4/CD5 fractions showed that the PF contains 22% more MSI2 than the IF, and the IF 13% more than the RF in all 25 CLL cases studied (Fig. S1D).

Hence, the highest amounts of MSI2 protein are found in the most recently divided CLL cells in patient blood.

### In vitro culture conditions that induce cell proliferation and mimic the in vivo state lead to enhanced MSI2 expression

Because CLL-cell proliferation occurs in the microenvironment of lymphoid tissues, presumably promoted by external signals [38–40], we tested MSI2 levels in leukemic cells from lymph node (LN) and PB samples. The paired analysis, different tissues from the same patient (*n* = 3), as well as the un-paired analysis, different tissues from different patients (*n* = 8), revealed that MSI2 is higher in LN than PB and also higher in the LN PF than RF (Figs. 1C, D and S1E). Interestingly, MSI2 levels in the PF from LN are higher than the PF from PB, and the same observations were seen when we compared IF or RF from each tissue (IF-LN > IF-PB and RF-LN > RF-PB; Fig. 1D).

**Fig. 2:**
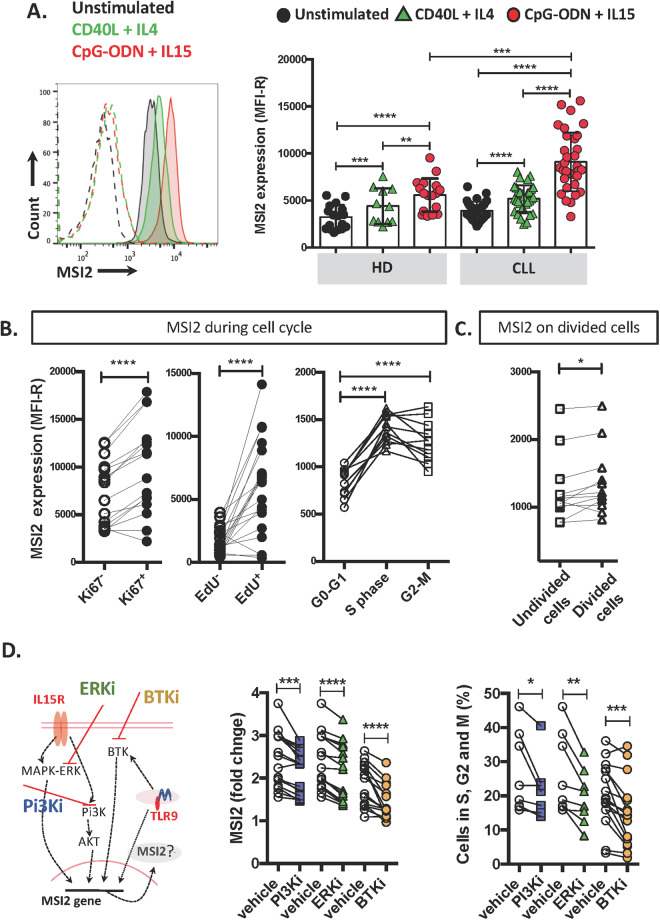
In vitro exposure of CLL cells to microenvironment-like signals results in increased MSI2 levels. **A** Representative flow cytometry histogram of MSI2 expression in CD19^+^CD5^+^ cells stimulated with CD40L + IL4 (green), CpGODN+IL15 (red), or not stimulated (black). The isotype control for each condition is shown as dashed lines. MSI2 protein in HD and CLL B cells (left). Black circles represent unstimulated cells, green triangles represent cells stimulated with CD40L + IL4, and red circles represent cells stimulated with CpG-ODN + IL15. **B** MSI2 expression in Ki67^+^ and Ki67^−^; in EdU^+^ and EdU^−^ cells and in different phases of the cell cycle. **C** MSI2 protein expression in undivided and divided CLL cells. Each circle/triangle/square represents one patient. **D** Representative signaling pathways of MSI2 expression in CLL cells. Leukemic cells were treated with CpG-ODN + IL15 for 3 days and then incubated for 24 h with PI3K inhibitor (LY294002, 50 µM) or ERK (SCH772984, 2 µM) or for 72 h with a BTK inhibitor (PCI 32765, 1 µM). MSI2 expression represented as fold change in CLL cells from 16 patients (PI3Ki, blue squares; ERKi, green triangles, and BTKi, orange circles) (middle panel). Percentage of B cells in S, G2, and M phases of the cell cycle after incubation with CpG-ODN + IL15 without and with the kinases inhibitors (left). Statistical analysis was performed using paired *t*-test analysis. Shown are individual values and mean ± SEM. *P < 0.05; **P < 0.01; ***P < 0.001; ****P < 0.0001.

To recapitulate this in vivo activation state, we cultured CLL PBMC from 38 patients with T-cell dependent (CD40L + IL4) and T-cell independent (CpG-ODN + IL15) [36] signals. Both sets of stimuli significantly increased MSI2 in CD19^+^CD5^+^ cells (Fig. 2A), with TLR9 + IL-15R stimulation upregulating MSI2 protein 2.82 ± 1.3-fold and CD40L + IL4 increasing MSI2 1.67 ± 0.9-fold (Fig. S2B). When we stimulated B cells from 17 HDs with the same stimuli, we found similar significant MSI2 upregulation (1.7 ± 0.4; 1.8 ± 0.45; Fig. S2A), although CpG-ODN + IL15 induced higher fold changes in leukemic than normal B cells (Fig. 2A).

Together, the results suggest that ex vivo and in vitro signals that induce B-cell growth lead to enhanced MSI2 protein expression in leukemic and normal B lymphocytes.

### Dividing and recently divided cells contain more MSI2 protein than resting CLL cells

Since MSI2 protein levels are naturally higher in the PF (PB and LN) (Fig. 1B–D) and in in vitro stimulated CLL cells (Fig. 2), we evaluated the extent that MSI2 levels differ in dividing and recently divided cells compared to undivided cells. To do so, we determined the levels of MSI2 in cells that entered the cell cycle (upregulation of Ki-67), replicated DNA (EdU incorporation), and were at different phases of the cell cycle (DAPI), as well as in recently divided cells (cell trace dilution) using flow cytometry. See Fig. S3 for the gating strategy for these studies.

CpG-ODN + IL15 stimulation of CLL cells led to more Ki67^+^ and EdU^+^ cells, more cells in S and G2-M phases, and more cells that completed all cell cycle phases and divided (Fig. S4A–D). Since CD40L + IL4 stimulation did not provide as strong a stimulus for DNA replication in CLL cells as CpG-ODN + IL15 (Fig. S4A, B), that condition was not evaluated here.

Ki67^+^ and EdU^+^ CLL cells displayed higher levels of MSI2 than Ki67^−^ and EdU^−^ cells (Fig. 2B). Similar results were recorded when we analyzed MSI2 levels in EdU-incorporating cells from the CLL-derived lines, MEC1 and OSU-CLL (Fig. S5A–H). Furthermore, MSI2 levels were higher in cells in S and G2-M (Fig. 2B) than G0–G1 phases of the cell cycle and in recently divided cells (Fig. 2C).

Since CpG-ODN + IL15 stimulation induces AKT, MAPK-ERK [36, 41], and BTK [42] signaling (Fig. S6A–D), we evaluated the roles of these pathways in MSI2 synthesis using pharmacologic inhibition. Blockage of AKT, ERK, and BTK signaling (Fig. S6E–G) led to significantly decreased MSI2 levels in a dose-dependent manner (Fig. S6H–J). PI3Ki and ERKi reduced MSI2 levels on average 0.88 ± 0.067 and 0.88 ± 0.06, and BTKi by 0.71 ± 0.1 compared to the effects of the vehicle control (Fig. 2D). In addition, each kinase inhibitor reduced significantly the number of cells in the S, G2, and M phases of the cell cycle (Fig. 2D).

In myeloid malignancies, HOXA9 upregulates MSI2, and MSI2 then downregulates NUMB, a negative regulator of NOTCH, thereby promoting cell division during blast crisis [43]. Moreover, HOXA9 is a direct target of MSI2 [18, 43]. Therefore, we investigated if this pathway was involved in regulating the growth of CLL cells. While CpG-ODN + IL15 stimulation increased MSI2 protein levels (Figs. 2B and S7A), there was no effect on HOXA9 levels (Fig. S7A). Moreover, knock-down of MSI2 did not affect HOXA9 and NUMB protein levels (Fig. S7B).

Together, these findings indicate that MSI2 protein levels are higher in dividing and recently divided cells and reducing CLL-cell proliferation by kinase inhibition decreases MSI2 levels. Notably, MSI2 upregulation occurs after activation of multiple signaling pathways but not by the HOXA9/MSI2/NUMB pathway.

### Downregulation of MSI2 protein leads to diminished CLL-cell survival

**Fig. 3:**
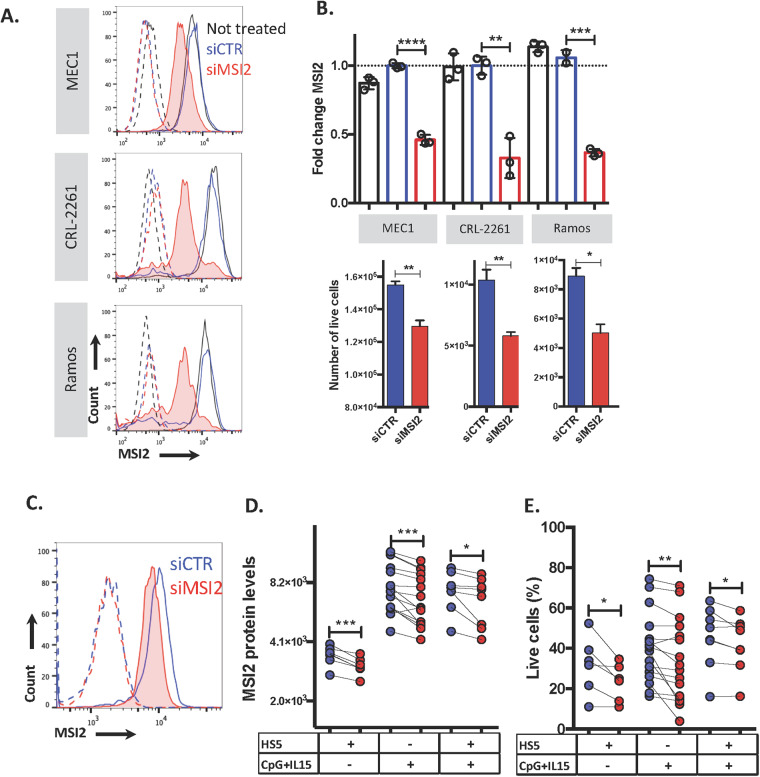
Downregulation of MSI2 in CLL cells leads to apoptotic cell death. **A** Representative flow cytometry histogram of MSI2 expression in MEC1, CRL-2261 (non-Hodgkin’s lymphoma), and Ramos (Burkitt’s lymphoma) untreated (black) or treated with siCTR (blue) or with siMSI2 (red). Dotted line is the isotype control for each condition. **B** MSI2 protein levels (siMSI2:siCTR) in each cell line in triplicate (upper panel). Number of viable cells in each cell line treated with siCTR (blue) or with siMSI2 (red) (lower panel). **C** Representative histogram of MSI2 expression in CD19^+^CD5^+^ cells treated with siCTR (blue) or siMSI2 (red). The isotype control for each condition is shown as dashed lines. **D** MSI2 expression in CLL cells treated with siCTR and siMSI2 in co-culture with HS5; stimulated with CpG-ODN + IL15; and stimulated with CpG-ODN + IL15 in co-culture with HS5. **E** Percentage of live cells after siRNA treatment. Paired *t*-test analysis was performed for each CLL patient. *P < 0.05; **P < 0.01; ***P < 0.001; ****P < 0.0001.

We next investigated the importance of MSI2 to CLL-cell survival. First, we performed MSI2 loss-of-function experiments in the CLL-derived MEC1 cell line, as well as cell lines from a non-Hodgkin’s lymphoma patient (CRL-2261) and a Burkitt’s lymphoma patient (Ramos) as B-cell controls. siMSI2 efficiently depleted MSI2 mRNA in MEC1 cells, using semi-quantitative and real-time qPCR (Figs. S8A, B). This led to a significant fall in flow cytometry-determined MSI2 protein levels (MEC1: 54%; CRL-2261: 67%; Ramos: 63%; respectively; Fig. 3A, B) and to a significant decrease in the number of the viable cells (MEC1: 13%; CRL-2261: 44%; Ramos: 43%; Fig. 3B).

Similarly, when we incubated the same siMSI2s with primary CLL cells co-cultured with or without HS5 feeder cells with or without CpG-ODN + IL15, each condition resulted in a significant MSI2 decrease in tumor-cell survival compared to the control (Fig. 3C–E).

Collectively, these findings indicate that MSI2 has an important role in CLL-cell survival.

### MSI2 knock-down in CLL cells induces cell cycle arrest and apoptosis by upregulating Caspase 3, p27kip1, and phospho-p53

**Fig. 4:**
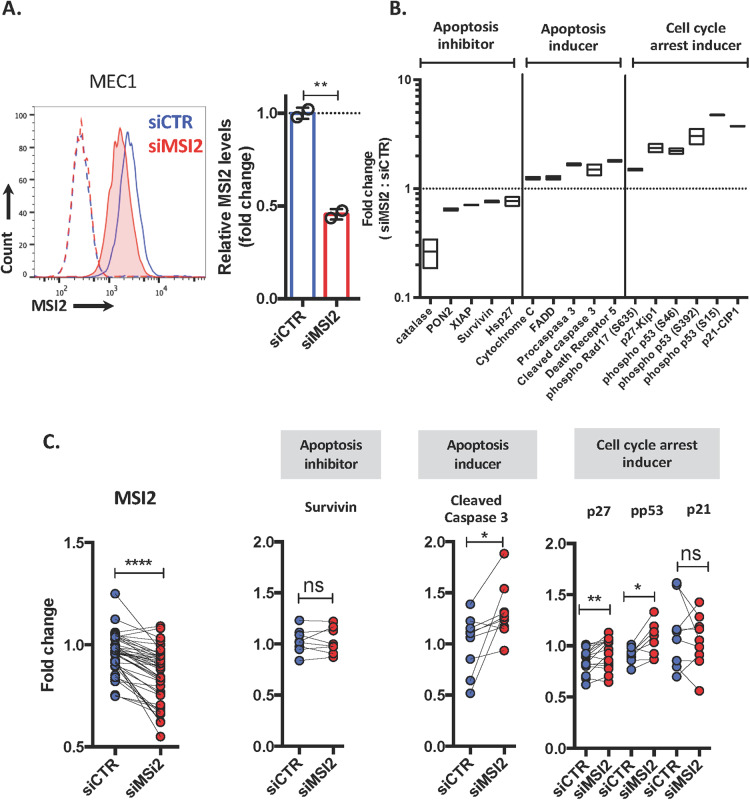
MSI2 knockdown induces apoptosis and cell cycle arrest by caspase 3, p27kip, and p53 signaling pathway. **A** Representative flow cytometry histogram of MSI2 expression in MEC1 cells treated with siCTR (blue) or with siMSI2 (red). Dotted line is the isotype control for each condition. MSI2 expression in MEC1 siCTR and siMSI2. **B** Relative apoptotic protein levels (siMSI2:siCTR) obtained from the array. Three groups of proteins were identified: apoptosis inhibitors; apoptosis inducers; and cell cycle arrest inducers. **C** MSI2, SURVIVIN, cleaved caspase, p27kip1, phosphor-p53, and p21Cip expression in CLL cells transfected with siMSI2 (red) or siCTR (blue). Paired *t*-test analysis was performed. *P < 0.05; **P < 0.01; ****P < 0.0001.

Since downregulation of MSI2 led to reduced CLL-cell survival, we determined the basis for this using a human apoptosis array and protein extracts from MEC1 cells treated with siMSI2 and siCTR (Fig. S9A). In addition to confirming significant downregulation of MSI2 protein levels (48% reduction; Fig. 4A), this revealed corresponding significant downregulation of the apoptosis-inhibitors, catalase (0.3-fold change), PON2 (0.6-fold change), XIAP (0.7-fold change), SURVIVIN (0.8-fold change) and Hsp27 (0.8-fold change) and significant upregulation of the apoptosis-inducers, cytochrome C (1.2-fold change), FADD (1.3-fold change), procaspase3 (1.7-fold change), cleaved caspase 3 (1.5-fold change) and death receptor 5 (1.8-fold change) (Figs. 4B and S9).

Furthermore, downregulation of MSI2-induced cell cycle arrest with a significant upregulation of phospho-Rad17 (1.5-fold change), p27kip1 (2.4-fold change), phospho-p53 (S46: 2.2-fold, S392: 3.0-fold, S15: 4.7-fold), and p21Cip1 (3.7-fold change) (Figs. 4B and S9 C, D).

To confirm these results in primary CLL cells, we quantified the abundance of SURVIVIN, cleaved caspase 3, p27kip1, phospho 53 (S15), and p21Cip1 in leukemic B cells stimulated with CpG-ODN + IL15 and treated with siMSI2 or siCTR. Downregulation of MSI2 in CLL cells significantly upregulated cleaved caspase 3 (1.3-fold), p27kip1 (1.1-fold), and phospho-p53 (1.2-fold) compared with siCTR. No differences were found in SURVIVIN and p21Cip1 levels (Fig. 4C).

Together, these results indicate that MSI2 knock-down induces cell cycle arrest and death of CLL cells.

### Specific blocking of MSI2 function eliminates CLL cells in vitro, in particular the dividing/recently divided B-cell fractions

**Fig. 5:**
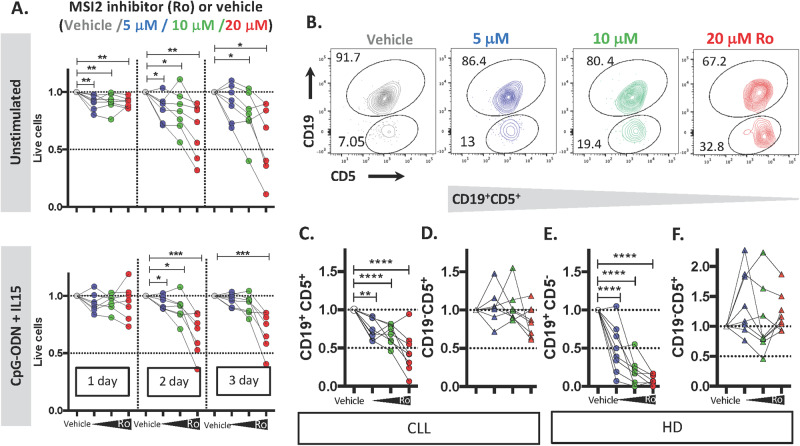
Inhibition of MSI2 function by locking mRNA binding induces cell death in CLL cells. **A** Viability of PBMC treated with MSI2 inhibitor, Ro 08-2750 (Ro). PBMCs from CLL patients were treated with 5, 10, and 20 μM of Ro along with CpG-ODN + IL15 stimulation (bottom panel) or without (upper panel) for 3 days. Flow cytometry results for day 1, 2, and 3 are shown in the Figure. Paired *t*-test analysis was performed using the percentage of viable cells from each patient culture after exposure to different doses of Ro compared to similar amounts of vehicle. *P < 0.05; **P < 0.01. **B** Representative flow cytometry profile of CD19 and CD5 surface expression highlighting the percentage of CD19^+^CD5^+^ and CD19^−^CD5^+^ (corresponding to CLL B cells and normal T cells from the same patients) after treatment with 5, 10, and 20 μM of Ro for 2 days. **C**–**F** Viable B and autologous T cells treated with 5 (blue), 10 (green), and 20 (red) μM of Ro. **C**, **D** show results with CLL samples and **E**, **F** are findings with HD samples.

Minuesa et al. have described a small molecule (Ro 08-2750) that binds selectively and directly to the RNA MSI2 binding site leading to loss of MSI2 function and affecting survival of myeloid leukemia cells [44, 45]. Therefore, we tested the effects of incubating varying concentrations of the drug (5, 10, and 20 μM) for 3 days on primary CLL PBMCs (*n* = 7 patients), either unstimulated or CpG-ODN + IL15-stimulated (Fig. 5A, upper and lower panels, respectively; Fig. S10A). Ro 08-2750 significantly reduced the number of viable cells compared with the vehicle control. The effect was time- and dose-dependent, reaching a maximum at 48 h, and the effects were similar for cells with or without stimulation (Fig. 5A).

Next, we tested the relative cellular specificity of Ro 08-2750, evaluating the number of viable leukemic B (CD19^+^CD5^+^) and normal autologous T (CD19^−^CD5^+^) cells in CLL patients after exposure in vitro. CLL PBMCs were incubated with the drug (5, 10, 20 μM) for 2 days, and the number of viable cells determined by flow cytometry using counting beads. There were significantly lower numbers of leukemic B cells (5, 10, or 20 μM: 25%, 30% and 55% reduction, respectively; Fig. 5B–D) and myeloid cells (granulocytes: 25%, 50% and 93% reduction; monocytes: 31%, 56% and 97%; Fig. S10B). Notably, T-cell numbers were unaffected at the lower drug concentrations (5 and 10 μM: 0% at both concentrations), and at the highest concentration (20 μM), there was only an insignificant 15% reduction in T-cell numbers compared to a 55% B-cell reduction. A similar restricted, greater effect of Ro 08-2750 on normal B cells than T cells was observed in HD PBMC samples (Fig. 5E, F). Thus, Ro 08-2750 preferentially and more effectively affects B cells than T cells.

**Fig. 6:**
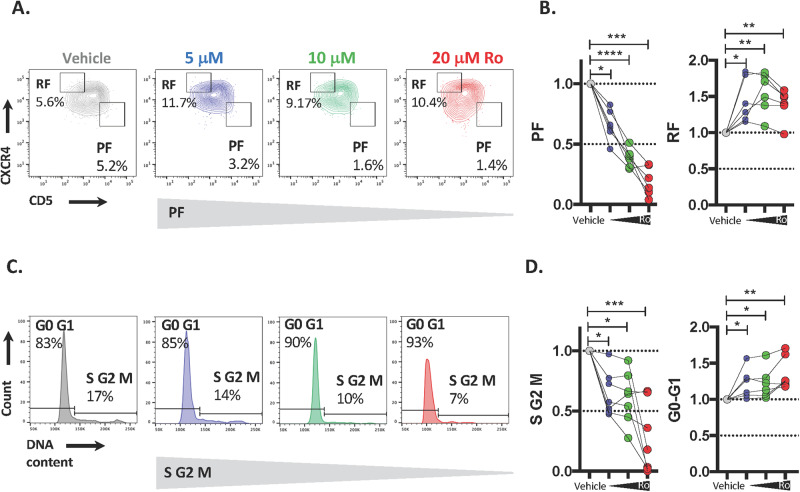
Inhibition of MSI2 by Ro 08-2750 reduces the number of dividing CLL cells. **A** Representative flow cytometry profile of CXCR4 and CD5 surface expression highlighting the effect of Ro on the PF and RF. **B** Viable relative PF (left) and RF (right) cells after treatment with Ro. **C** Representative flow cytometry of DNA content to determine the percentage of cells in the different phases of the cell cycle. **D** Viable relative cells in S–G2–M phases of cell cycle (left) and G0–G1(right) after treatment with Ro.

Since we found higher levels of MSI2 in dividing cells and in their progeny, we tested whether the MSI2 inhibitor mainly affected proliferating cells. CpG-ODN + IL15-stimulated CLL cells were exposed to the drug for 2 days and then the percentage of proliferating cells, based on the number of cells in S, G2, and M phases of the cell cycle, and of recently divided cells, defined by CXCR4 and CD5, was evaluated. Interestingly, there was a dramatic, significant dose-dependent reduction of both the cycling (S–G2–M phases of cell cycle) and recently divided (CXCR4^Dim^CD5^Bright^) cells as well as an increase of resting cells (G0–G1 and CXCR4 ^Bright^ CD5^Dim^) (Figs. 6A–D and S10C, D).

Since MSI2 knockdown by siRNA induced cell cycle arrest and apoptosis by upregulating p27kip1 and Caspase 3 (Fig. 4), we determined if the same occurred after inhibition of MSI2 function by Ro 08-2750. MSI2 inhibition increased cleaved Caspase 3 and p27kip1 after 24 h of treatment (Fig. S11A), and these effects were not found at 72 h. Notably, both siRNA and Ro 08-2750 treatments upregulated active Caspase 3 as well as p27kip1 at 72 h (Fig. S11B). The MSI2 inhibitor had a more rapid effect on apoptosis than the siRNA, since these occurred within 24 h of treatment, whereas the siRNA required ~72 h. Both siRNA and Ro 08-2750 induced cell cycle arrest after 24 h as measured by upregulating p27kip1.

Hence, rapid and delayed elimination of MSI2 function deletes CLL cells in a dose-dependent manner, with a preferential action on cycling and recently divided leukemic B cells. In addition, inhibition of MSI2 has a more significant effect on myeloid cells. In contrast, normal T cells are spared.

### Pharmacological inhibition of MSI2 in a CLL mouse model reduces tumor burden

**Fig. 7:**
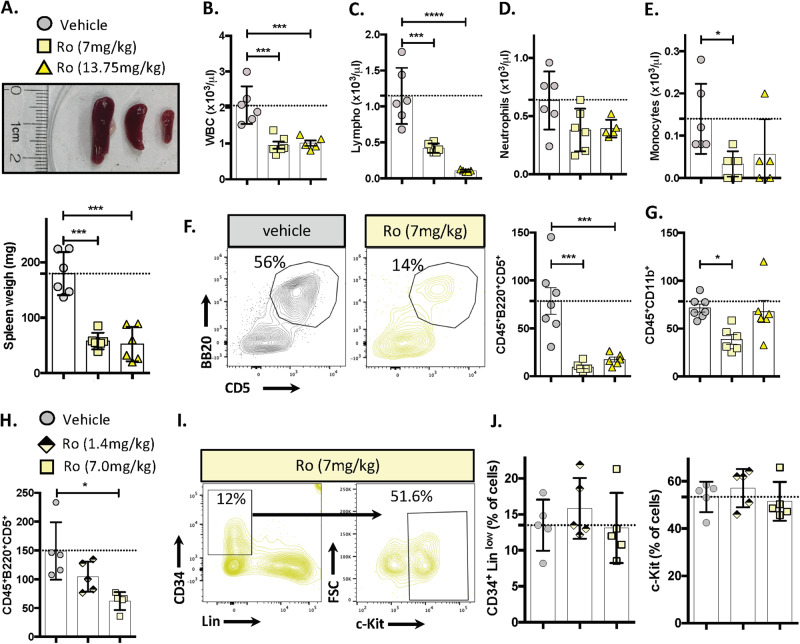
Inhibition of MSI2 function in murine CLL cells significantly reduces tumor burden. In vivo Ro 08-2750 treatment was evaluated in an adoptive transfer mouse CLL model. TCL1-192 cells were injected into SCID mice, and recipient mice were given either vehicle (DMSO, gray circle) or 1.4 mg/kg (black and light-yellow diamond), 7 mg/kg (yellow square) or 13.75 mg/kg (bright yellow triangle) doses twice a week. Mice were sacrificed for analysis after 19 days of treatment. **A**. Spleen weights in mgs at time of sacrifice. Each data point represents an individual vehicle- or Ro-treated mouse. **B** White blood cell (WBC, (×10^3^/μL)), **C** lymphocyte (×10^3^/μL), **D** neutrophils (×10^3^/μL), **E** monocytes (×10^3^/μL) counts at time of sacrifice. **F** Representative flow cytometry profile from PB mice CLL cells (B220^+^CD5^+^) treated with vehicle (gray) and Ro (yellow) and CD45^+^B220^+^CD5^+^ cell count on the left. **G** CD45^+^CD11b^+^ cell count on the far left. **H** PB CD45^+^B220^+^CD5^+^ cell counts on mice treated with 1.4 mg/kg (black and light-yellow diamond) or 7 mg/kg (yellow square). **I** Representative flow cytometry profile of bone marrow HSC, multi and oligopotent cells (CD34^+^Lin^low^ and CD34^+^Lin^low^c-Kit^+^) for Ro (yellow) treated mice. **J** Percentage of CD34^+^Lin^low^ and CD34^+^Lin^low^c-Kit^+^. Each data point represents an individual treated mouse. Unpaired *t*-test was performed. *P < 0.05; **P < 0.01; ****P < 0.0001. None of the HSC, multi and oligopotent cells comparisons (**J**) indicated a significant (*P* < 0.05) result.

Finally, we determined Ro 08-2750’s efficacy in an in vivo aggressive murine CLL leukemia model [46]. TCL1-192 cells were intravenously injected into SCID mice, and 4 days later the animals were treated with Ro 08-2750 at 13.75 or 7.0 mg/kg intraperitoneally twice a week for 21 days. Notably, both treated groups experienced a significant reduction in spleen weights and in murine CD45^+^ cells, total white blood cells, lymphocytes, and leukemic B lymphocytes (CD45^+^B220^+^CD5; Fig. 7A–F). As in AML-bearing mice [45], Ro 08-2750 administration was well tolerated, with no or insignificant declines in red blood cells, hemoglobin levels, mean corpuscular volumes, platelets (PLT) (Fig. S12A-D) and neutrophils (Fig. 7D) in treated mice. Monocytes were reduced in treated animals (Fig. 7E, G).

In order to find the lowest dose needed to have a significant effect on CLL cells in vivo and to test the effect of MSI2 inhibition on early hematopoietic cells, we next treated recipient animals with the previous low Ro 08-2750 dose, 7.0 mg/kg, and with a lower dose 1.4 mg/kg. Only mice receiving 7 mg/kg had significantly decreased numbers of B220^+^CD5^+^ leukemic cells, although a trend was found with the lowest dose (Fig. 7H). Additionally, there was not a significant change in the percentage of early hematopoietic cells (CD34^+^Lin^low^ or CD34^+^Lin^low^c-Kit^+^) in the bone marrows of leukemia-bearing mice with both Ro 08-2750 doses (Fig. 6I). Correspondingly, no changes were observed in any of the HSCs progeny of the erythrocytic or megakaryocytic lineages (Fig. S12E-H).

To more directly determine the effects of Ro 08-2750 on HSCs and progenitor cells, we evaluated the percentage of stem (HSC, MPP1, MPP2, MPP4) and progenitor (CMP, GMP, MEP) cells from bone marrow of SCID mouse transplanted with TCL1-192 and treated with 7 mg/kg of Ro 08-2750. No differences in any of these populations were found for drug-treated compared to vehicle-treated animals (Fig. S13).

Additionally we determined the effect of MSI2 inhibitor on HSCs and progenitors from CLL patients and from age-matched people undergoing hip replacement for degenerative arthritis. CD34^+^ enriched cells (hematopoietic cells and progenitors, HSPCs) from both sources were treated with or without Ro 08-2750 (10 μM) and colony forming units were scored 14 days later. The MIS2 inhibitor did not affect hematopoiesis by HD nor CLL HSPCs cells (Fig. S14A, B). The only significant change detected was an increase in BFU-E in one CLL patient after drug exposure.

Together these results indicate that MSI2 inhibition in mouse and man selectively reduces B-cell leukemia burden and mature myeloid cells, while not affecting HSCs or non-lymphoid/myeloid progenitors.

### MSI2 expression is the highest in poor outcome CLL patients

**Fig. 8:**
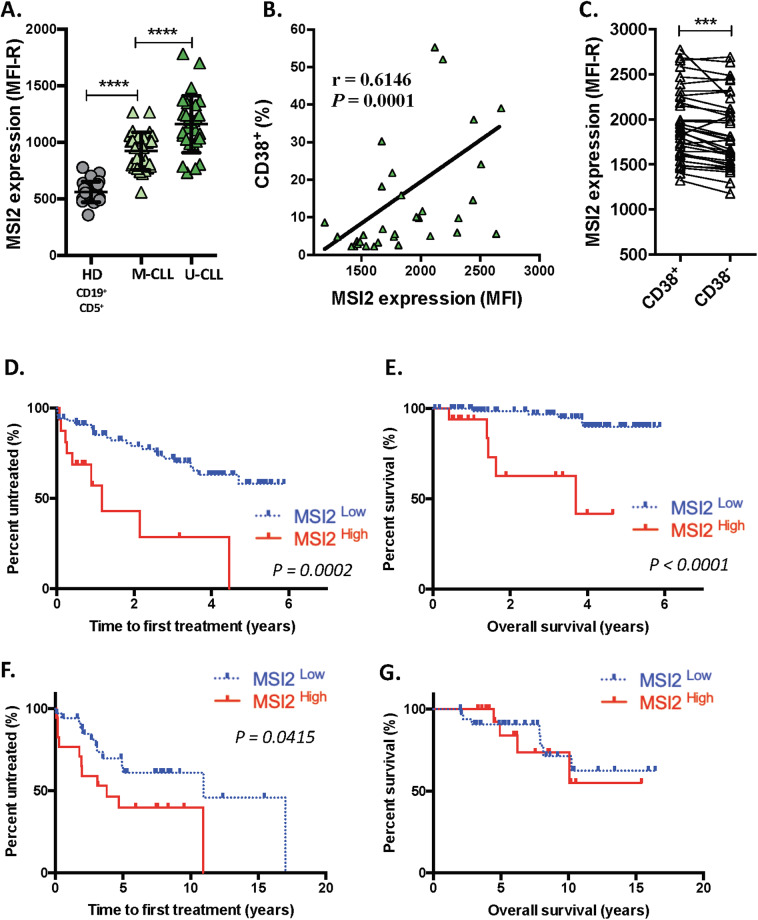
High MSI2 levels correlate with poor clinical course and outcome in CLL. **A** MSI2 expression in CD19^+^CD5^+^ B cells from M-CLL and U-CLL patients compared to CD19^+^CD5^+^ HD B cells. Statistical analysis was performed using One-way ANOVA, Tukey’s multiple comparison test. Shown are individual values and mean ± SEM. *P < 0.05; **P < 0.01; ***P < 0.001; ***P < 0.0001. **B** Correlation between CD38 and MSI2 expression in CLL B cells (Spearman *r*, *r* = 0.6146, *P* = 0.0001). **C** MSI2 protein levels in CD38^+^ and CD38^−^ cells in the same CLL clones. **D**–**G** Kaplan–Meier curves for time-to-first-treatment (TTFT) and overall survival (OS) in CLL patients with higher or lower MSI2 mRNA levels (*n* = 111, Table S1B, D, E) and protein levels (*n* = 54, Table S1A, F–G). Patients in **D**, **E** (*n* = 111) and in **F**, **G** (*n* = 57) are distinct cohorts that were dichotomized into high and low MSI2 levels (mRNA and protein) using maxstat package for R-2.8.0. MSI2^high^ exhibited significantly shorter TTFT at protein and mRNA level (long-rank test, *P* = 0.0002 and 0.0415, respectively). MSI2^high^ exhibited significantly shorter OS at mRNA level (long-rank test, *P* < 0.00001).

Previous studies suggest that MSI2 is one of a series of genes associated with worse clinical outcomes in CLL [27, 31, 32]. Moreover, since our GEP studies indicated that MSI2 mRNA was higher in U-CLL than M-CLL and highest in the PF of U-CLL cases (Fig. S1B, C), we analyzed MSI2 protein levels in these CLL and intraclonal subtypes. MSI2 levels were higher in 26 U-CLL than 29 M-CLL patients (Table S1A), and the levels in each CLL type were greater than in normal B cells from 25 HDs (Fig. 8A). Moreover, MSI2 levels correlated with the percentage of CLL cells displaying the poor prognostic marker CD38 (Fig. 8B). Additionally, MSI2 levels were higher in CD38^+^ than CD38^−^ leukemic cells from the same CLL patient (Fig. 8C), consistent with CD38^+^ being enriched in recently divided cells [34, 35].

Based on these findings and on MSI2 levels being required for cell proliferation and MSI2 downregulation or functional blocking inducing cell death, we asked if MSI2 mRNA or protein levels correlated with patient clinical course and outcome in untreated patients from two different centers (Table S1A, B). First, TTFT and OS were correlated with MSI2 mRNA levels in 111 patients (Table S1B). This indicated that patients with higher MSI2 expression had shorter TTFTs and OS (Fig. 8D, E). Next, using a different cohort of patients (*n* = 57; Table S1A), we found that higher MSI2 protein levels associated with shorter TTFT (*P* = 0.0415; Fig. 8F); there was no difference in OS (Fig. 8G), possibly because only 11 of the 57 patients analyzed expired.

To address the question whether MSI2 is an independent prognostic indicator, we performed a multivariate Cox proportional hazards model analysis using the 111 patient cohort (Table S1B). In univariate analysis, Rai stage, IGHV mutational status, CD38 percentage, and MSI2 levels were significantly different for TTFT and OS (Table S3A). However, multivariate analysis showed that only Rai stage and MSI2 levels were statistically significant independent risk factors for OS of CLL patients (Table S3B and Fig. S15).

Thus, MSI2 mRNA and protein expression predict worse clinical courses and outcomes in CLL.

## Discussion

The data reported here provide new information about the role of MSI2 protein in the growth and survival of CLL cells. Support for MSI2 promoting CLL-cell growth comes from our ex vivo finding that MSI2 expression is upregulated in recently divided CLL cells and our in vitro studies of leukemic cells induced to divide by signals resembling those delivered in the tissue microenvironment where CLL-cell birth occurs [40]. These signals are likely relevant in patients because TLR9 and IL15R are expressed by CLL-cells [47–49]; TLR9 signaling is a strong activator of AKT and ERK in CLL cells [41] and of BTK in normal B cells [42]; and IL15, which is constitutively produced by stromal cells in bone marrow, spleen, and LN [50–52], promotes the growth of leukemic B cells [49, 50].

Our knockdown studies and in vitro and in vivo MSI2 functional blocking studies using a small molecule inhibitor (Ro 08-2750) that binds selectively to the RNA MSI2 binding site [44, 45] support the idea that MSI2 promotes CLL-cell survival. Specifically, downregulation of MSI2 in primary CLL cells and in three B-cell lines (MEC1, CRL-2261, and Ramos) reduced cell viability, suggesting MSI2 plays a role in CLL-cell and B-cell lymphoma-cell survival. Consistent with this was the concomitant upregulation of cleaved caspase 3 and the tumor suppressors p27kip1 [53] and phospho-p53 [54] protein levels. Furthermore, specific MSI2 inhibition reduced the numbers of proliferating and recently divided leukemic B cells in vitro and diminished spleen weight and WBC and B-cell counts in vivo. In keeping with our findings, MSI2 loss-of-function or silencing abrogates cell growth and induces apoptosis in several tissues [20, 55, 56] and in AML and CML cell lines [10, 44, 57] as well as in primary AML cells [58].

In other settings, MSI2 plays an important role regulating gene expression by repressing [14, 15] or inducing [16–18] protein translation. These divergent actions complicate studies of MSI2 function and the pathways MSI2 regulates. For example, a study modeling chronic and blast crisis CML in mice proposed that Nup98-HOXA9 triggers MSI2 expression that in turn represses NUMB and induces blast crisis [43]. However, our studies of expression and relationships of HOXA9, MSI2, and NUMB with MSI2 did not indicate a causative role for HOXA9 in MSI2 levels; furthermore, increasing MSI2 levels in vitro did not affect HOXA9 levels. Also, we did not observe changes in NUMB or HOXA9 levels in knock-down MSI2 B cells from CLL patients. Together these results suggest that a HOXA9/MSI2/NUMB pathway is not responsible for CLL-cell proliferation, and identifies control of MSI2 action as a productive avenue of research for the future. Somewhat similar results have been found for pancreatic β cells, in which MSI2-induced cell proliferation is NUMB-independent [59]. However, we did find that blocking the AKT, ERK, or BTK signaling pathways in primary CLL cells reduced MSI2 protein levels, indicating that multiple signaling pathways control MSI2 expression. This strongly suggests that MSI2 is critical for multiple biologic processes, in particular in leukemic B cells.

The critical roles for MSI2 in CLL-cell survival and growth likely relate to the higher MSI2 levels in CLL cells expressing poor outcome prognostic markers, i.e., unmutated IGHV and elevated numbers of CD38^+^ cells [27, 31, 32], and experiencing shorter TTFT and OS. These findings are also consistent with MSI2 associating directly with tumorigenesis and poor prognosis in other tumor types [10, 17, 20–22, 24–30, 55].

Collectively, our documentation that MSI2 affects CLL-cell growth and survival and correlates with worse clinical course and outcome suggests that this molecule and its pathway are potential therapeutic targets. This conclusion is supported by the in vitro and in vivo effects of MSI2 inhibition by Ro 08-2750. Especially provocative is finding that the drug mainly affects B cells and myeloid cells but spares normal autologous T cells, HSCs, and their erythrocyte and PLT progeny. Furthermore, the apparent preferential action of the drug on cycling and recently divided CLL cells suggests that selective MSI2 inhibition would prevent disease progression since these are the fractions of leukemic clones that express the DNA mutator, AID, and hence could develop new DNA abnormalities that might lead to clonal evolution, disease advancement, and clinical deterioration. Thus, our findings suggest specific MSI2 inhibition as a potential treatment for CLL.

## Supplementary information

Supplemental Figure Legends

Supplementary Materials and Methods

Table S1A

Table S1B

Table S2

Table S3
