# Supplementary material for: Musashi 2 influences chronic lymphocytic leukemia cell survival and growth making it a potential therapeutic target
Source: Leukemia. 2021 Jan 27;35(4):1037–52. doi: 10.1038/s41375-020-01115-y (PMC8024198; doi:10.1038/s41375-020-01115-y)
Supplement: Supplementary file 1 — Supplemental Figure Legends [file 41375_2020_1115_MOESM1_ESM.pdf]

Supplementary figures

Figure S1: ***Higher MSI2 levels in CLL B cells and in recently-divided CLL cells.*** RNA was purified from B cells of 26 CLL ($CD5^+CD19^+$), and 11 HD ($CD19^+CD5^-$), and gene expression was performed using Illumina HumanHT12 beadchips. Microarray data were normalized using quantile normalization by GenomeStudio software (Illumina). MSI2 mRNA levels were evaluated by gene expression profile from **(A)** HD (n=11) and CLL (n=26) isolated B cells samples, **(B)** PF and RF from CLL B cells samples and **(C)** M and U-CLL patients. **D.** Relative MSI2 protein ratios (PF:IF, IF:RF, and PF:RF) in CLL B cells. **E.** MSI2 protein levels (MFI-R) in $CD19^+CD5^+$ cells from pair samples (n=3) PB and LN were obtained within one month. MSI2 expression (MFI-R) in each CXCR4/CD5 fraction (left). Statistical analysis was performed using One-way ANOVA, Tukey's multiple comparison test. Shown are individual values. * indicates $P<0.05$; ** $P<0.01$; *** $P<0.001$; *** $P<0.0001$.

Figure S2: ***In vitro microenvironment-like signals increase MSI2 levels.*** MSI2 expression in B cells from HD ($CD19^+CD5^-$, **A**) and CLL cells ($CD19^+CD5^+$, **B**) cells stimulated with CD40L+IL4 (green), CpGODN+IL15 (red), or not stimulated (black). Black circles represent unstimulated cells, green triangles represent cells stimulated with CD40L+IL4, and red circles represent cells stimulated with CpG-ODN+IL15. Each circle/triangle/square represents one patient. * indicates $P<0.05$; ** $P<0.01$; *** $P<0.001$; *** $P<0.0001$.

Figure S3: ***Gating strategy for analysis of CLL cells after in vitro stimulation.*** **A.** Single cells were gated based on forward and side scatter, and live cells selected using the Live and Dead reagent (ThermoFisher). Gates were set to include $CD19^+CD5^+$ cells and exclude T cells ($CD19^-CD5^+$) for the analysis of Ki-67 expression (**B**), incorporation of EdU (**C**), percentages of cells in the cell cycle (**D**), and dividing cells (**E**).

Figure S4: ***TLR9 + IL15 stimulation effectively induces proliferation of CLL B cells.*** Percentages of Ki-67^+ (**A**) and EdU^+ (**B**) cells after 4 days of *in vitro* stimulation with CD40L+IL4 or CpG-ODN+IL15. Black circles represent unstimulated cells; green triangles represent cells stimulated with CD40L+IL4; and red circles represent cells

stimulated with CpG-ODN+IL15. **C.** Percentage of cells in the G0-G1, S, and G2-M stages of the cell cycle in cells stimulated or not for 6 days with CpG-ODN+IL15. **D.** Representative flow cytometry histogram of B-cell division assessed by cell trace dye dilution after activation with CpG-ODN+IL15 for 6 days. Percentage of dividing cells in unstimulated or CpG-ODN+IL15 stimulated cell cultures. ** indicates *P*<0.01; *** *P*<0.001; **** *P*<0.0001

Figure S5: ***Dividing MEC1 and OSU-CLL cells express higher levels of MSI2.*** Representative histograms of MSI2 expression levels in live MEC1 **(A)** and OSU-CLL **(E)** cells are shown. Histograms of MSI2 expression in EdU^+ and EdU^- cells in MEC1 **(B and C)** and in OSU-CLL **(F and G)**. MSI2 protein levels in EdU^+ and EdU^- MEC1 cells (in triplicate) (**D**) and OSU-CLL (in triplicate) **(H).** * indicates *P*<0.05; ** *P*<0.01.

Figure S6: ***Effects of TLR9 + IL15 stimulation on phosphorylation of AKT, MAPK-ERK, and BTK.*** **A.** Representative signaling pathways of MSI2 expression in CLL cells. Phospho-AKT (T308) **(B)**, -MAPK-ERK (T202/204) **(C)** and -BTK (Y551/511) **(D)** levels in CLL cells stimulated (red circles) or not (open circles) with CpG-ODN+IL15 for 3 days. After stimulation, cells were incubated for 24 hrs. with PI3Ki (LY294002: 30, 40 and 50μM), ERKi (SCH772984: 0.02, 0.2 and 2μM) or 72 hrs. with BTKi (PCI 32765: 0.5, 1, 2μM). Relative phospho -AKT (T308) (PI3Ki:Vehicle) **(E)**, phospho MAPK-ERK (ERKi:Vehicle) **(F)** and phospho BTK levels (BTKi:Vehicle) **(G)** in CLL cells treated with kinase inhibitors. Relative MSI2 levels were evaluated with different concentrations of the kinase inhibitors and shown in relation to the vehicle **(H-J)**. * indicates P<0.05; ** *P*<0.01; *** *P*<0.001; **** *P*<0.0001

Figure S7: ***Lack of a role for the HOXA9/MSI2/NUMB pathway in CLL.*** **A.** Relative MSI2 and HOXA9 protein levels in CLL cells stimulated vs with CpG-ODN + IL15. **B.** MSI2, NUMB and HOXA9 relative protein levels after MSI2 knockdown in CLL B cells (siMSI2) compared with the negative control (siCTR). * indicates P<0.05; ** *P*<0.01; **** *P*<0.0001.

Figure S8*: Downregulation of mRNA MSI2 in MEC1 using small inhibitory RNAs.* mRNA MSI2 levels for MSI2 and β-actin were determined by semi-quantitative PCR **(A)**

and real time PCR **(B)** in MEC1, untreated cells, treated with negative siRNA control (siCTR) and with MSI2 siRNA (siMSI2) per duplicates. * indicates P<0.05; ** *P*<0.01.

Figure S9: ***Downregulation of MSI2 in MEC1 cells by siRNAs increases apoptosis-associated molecules.*** **A.** Human apoptosis array strategy. **B.** Survivin expression (apoptosis inhibitor) and cleaved caspase3 (apoptosis inducer) in MEC1 cells transfected with siMSI2 (red) or siCTR (blue). **C.** Expression levels of p27kip1, phospho p53 (S46, S392 and S15), and p21Cip1 in MSI2-downregulated cells (red) versus the control (blue). **D.** List of proteins differentially express in MSI2 downregulated MEC1 cells. * indicates P<0.05; ** *P*<0.01; **** *P*<0.0001.

Figure S10: Ro 08-2750 ***reduces the viability of CLL cells in vitro.*** **A.** PBMCs from CLL patients were treated with 5, 10 and 20 μM of Ro 08-2750 (Ro) along with (bottom panel) or without (upper panel) CpG-ODN+IL-15 stimulation for 3 days. Flow cytometry results for day1, 2 and 3 are shown in the Figure. **B.** Viable $CD19^{+}CD5^{+}$, $CD19^{-}CD5^{+}$, $CD19^{-}CD5^{-}CD11b^{+}$ and $CD19^{-}CD5^{-}CD16^{+}$ cells after treating CLL PBMCs with 5, 10 and 20 μM of Ro for 1 day. Percentage of viable cells in the PF, RF subset **(C.)** and viable cells in S-G2-M and G0-G1 phases of the cell cycle after treatment during 2 days with different concentrations of Ro **(D.)**. Statistical analysis is shown in the main figure.

Figure S11: ***Blocking MSI2 mRNA binding induces cell cycle arrest and apoptosis.*** PBMCs from CLL patients were treated with 10 and 20 μM of Ro 08-2750 (yellow) or siRNA (siCTR, blue or siMSI2, red). Relative cleaved caspase 3 (upper panel) and p27kip1 (lower panel) CLL B cells expression after 24hrs **(A.)** or 72 hrs **(B.)** of treatment.

Figure S12: ***Effects of Ro 08-2750 on murine CLL cells transferred into syngeneic immune deficient mice***. **A.** Scheme of *in vivo* Ro 08-2750 (Ro) treatment in an adoptive transfer mouse CLL model. TCL1-192 cells were injected into SCID mice, and after 4 days recipient mice were given intraperitoneally (i.p) either vehicle (DMSO, grey circle) or 1.4 mg/kg (black and light-yellow diamond), 7mg/kg (yellow square) or 13.75 mg/kg (bright yellow triangle) doses at days 1, 5, 8, 12 and 15. At 19 days of treatment, mice were sacrificed for analysis. **B.** Red blood cell (RBC) counts ($x10^{6}$/μL), **C.** hemoglobin levels (HGB, g/dL), **D.** mean corpuscular volume (MCV, fL) and **E.** platelets (PLT)

(x10^3/µL) counts at time of sacrifice from mice treated with vehicle (grey circles), Ro 7mg/kg (yellow squares) or 13.75 mg/kg (bright yellow triangles). RBC, HGB, MCV, and PLT counts were also evaluated at time of sacrifice in another experiment using Ro 1.4mg/kg (black and yellow diamonds) or Ro 7mg/kg (yellow squares). Each data point represents an individual treated (vehicle or Ro) mouse. Unpaired t-test was performed. None of the comparisons indicated a significant ($P<0.05$) result.

Figure S13: ***Effects of Ro 08-2750 on murine stem and progenitors cells.*** Ro 08-2750 effect on stem and progenitors cells *in vivo* in an adoptive transfer mouse model. TCL1-192 cells were injected into SCID mice and recipient mice were given either vehicle (DMSO, grey circle) or 7mg/kg (yellow circle) of Ro 08-2750 (**Figure S12A**). Fresh bone marrow cells were evaluated for stem cells and progenitors. **A.** Gating strategy. **B.** Percentage each group of subpopulations. Each data point represents an individual treated mouse. None of the stem cells and indicated a significant ($P<0.05$) result.

Figure S14: ***Effects of*** **Ro 08-2750** ***on human hematopoiesis.*** Colony forming unit assay on $CD34^+$ enriched cells (hematopoietic cells and progenitors, HSPCs) isolated from the bone marrows of CLL patients and age-matched healthy donors, treated with or without Ro 08-2750 (10µM). No significant differences were found.

Figure S15: ***Estimated hazard ratios (Cox regression) of four poor prognostic markers.*** Hazard ratio (HR) >1.0 indicate poor outcome. Multivariate analysis suggested that MSI2 expression and Rai stage are an independent prognostic marker of the OS and CD38 and Rai stage are independent markers for TTFT.

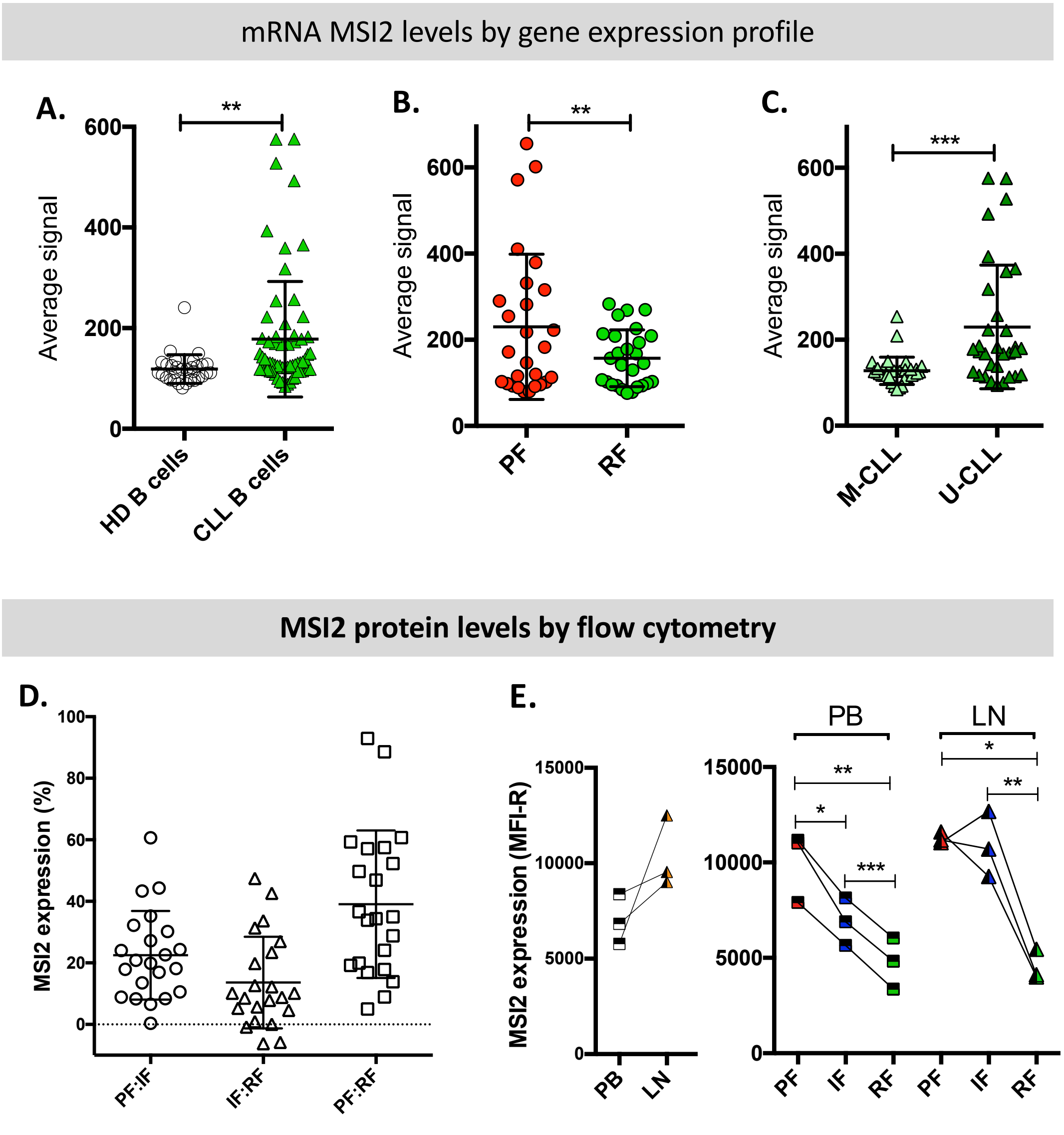
mRNA MSI2 levels by gene expression profile
A.
**
Average signal
0
200
400
600
HD B cells
CLL B cells
B.
**
Average signal
0
200
400
600
PF
RF
C.
***
Average signal
0
200
400
600
M-CLL
U-CLL
MSI2 protein levels by flow cytometry
D.
MSI2 expression (%)
0
20
40
60
80
100
PF:IF
IF:RF
PF:RF
E.
MSI2 expression (MFI-R)
0
5000
10000
15000
PB
LN
PB
LN
*
**
*
***
*
**
PF
IF
RF
PF
IF
RF

Figure S1

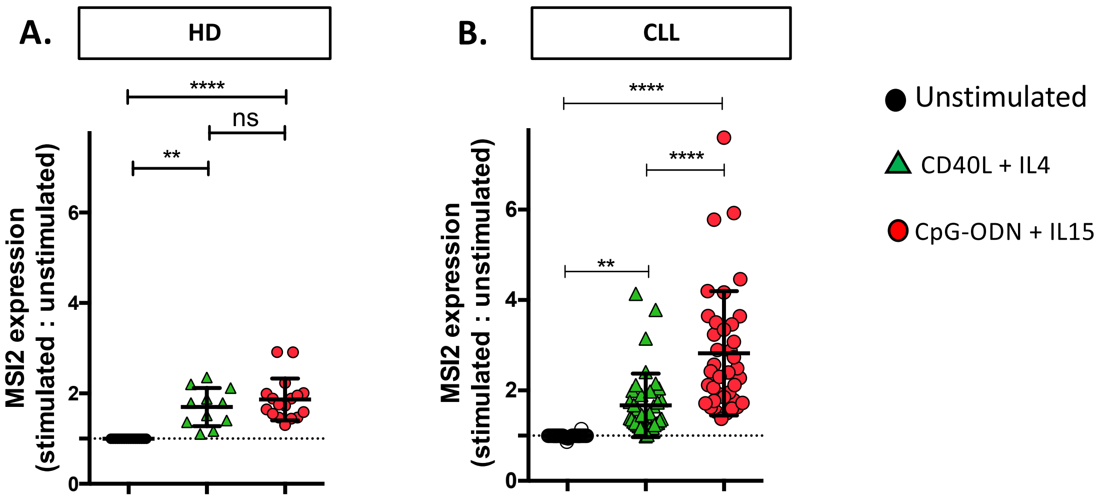
A.
HD
B.
CLL
MSI2 expression
(stimulated : unstimulated)
****
ns
**
****
****
**
0
2
4
6
Unstimulated
CD40L + IL4
CpG-ODN + IL15

Figure S2

Gating strategy

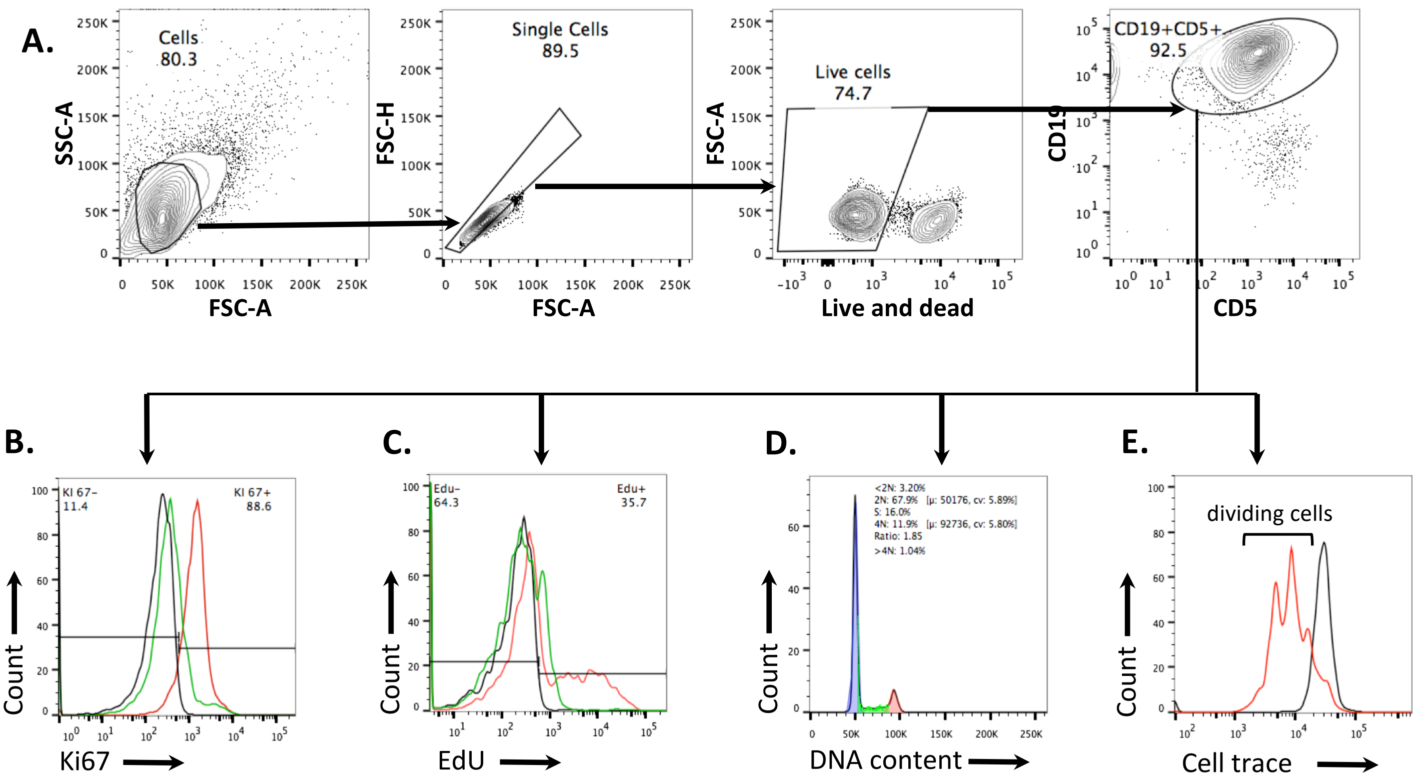

Black: Unstimulated
Green: CD40L+IL4
Red: CpG-ODN+IL15

G0-G1/ S phase/ G2-M

Unstimulated
CpG-ODN+IL15

Figure S3

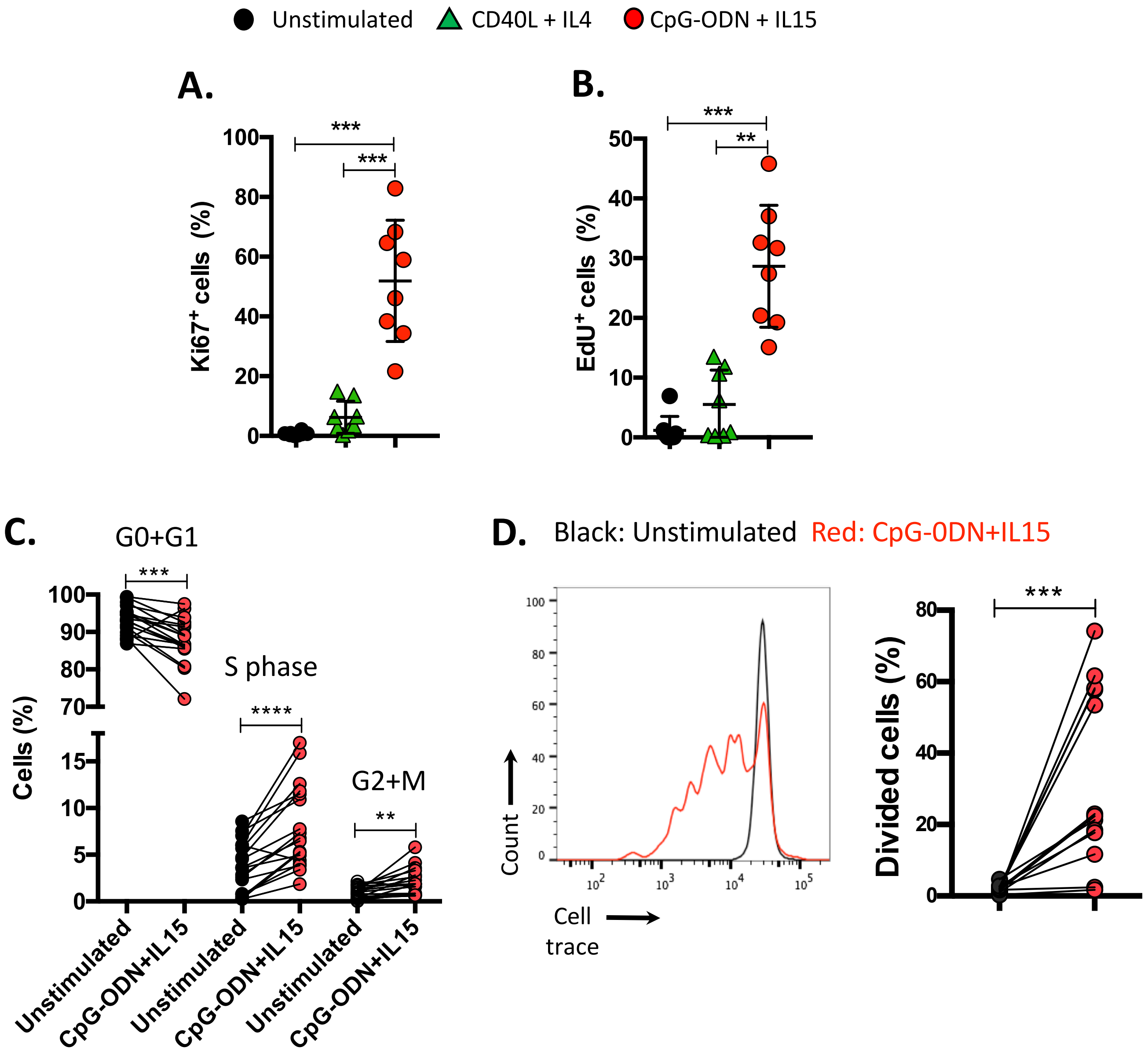
Unstimulated
CD40L + IL4
CpG-ODN + IL15
A.
Ki67+ cells (%)
***
***
100
80
60
40
20
0
B.
EdU+ cells (%)
***
**
50
40
30
20
10
0
C.
G0+G1
***
S phase
****
G2+M
**
Cells (%)
100
90
80
70
15
10
5
0
Unstimulated
CpG-ODN+IL15
Unstimulated
CpG-ODN+IL15
Unstimulated
CpG-ODN+IL15
D.
Black: Unstimulated
Red: CpG-0DN+IL15
Count
Cell trace
Divided cells (%)
***
80
60
40
20
0

Figure S4

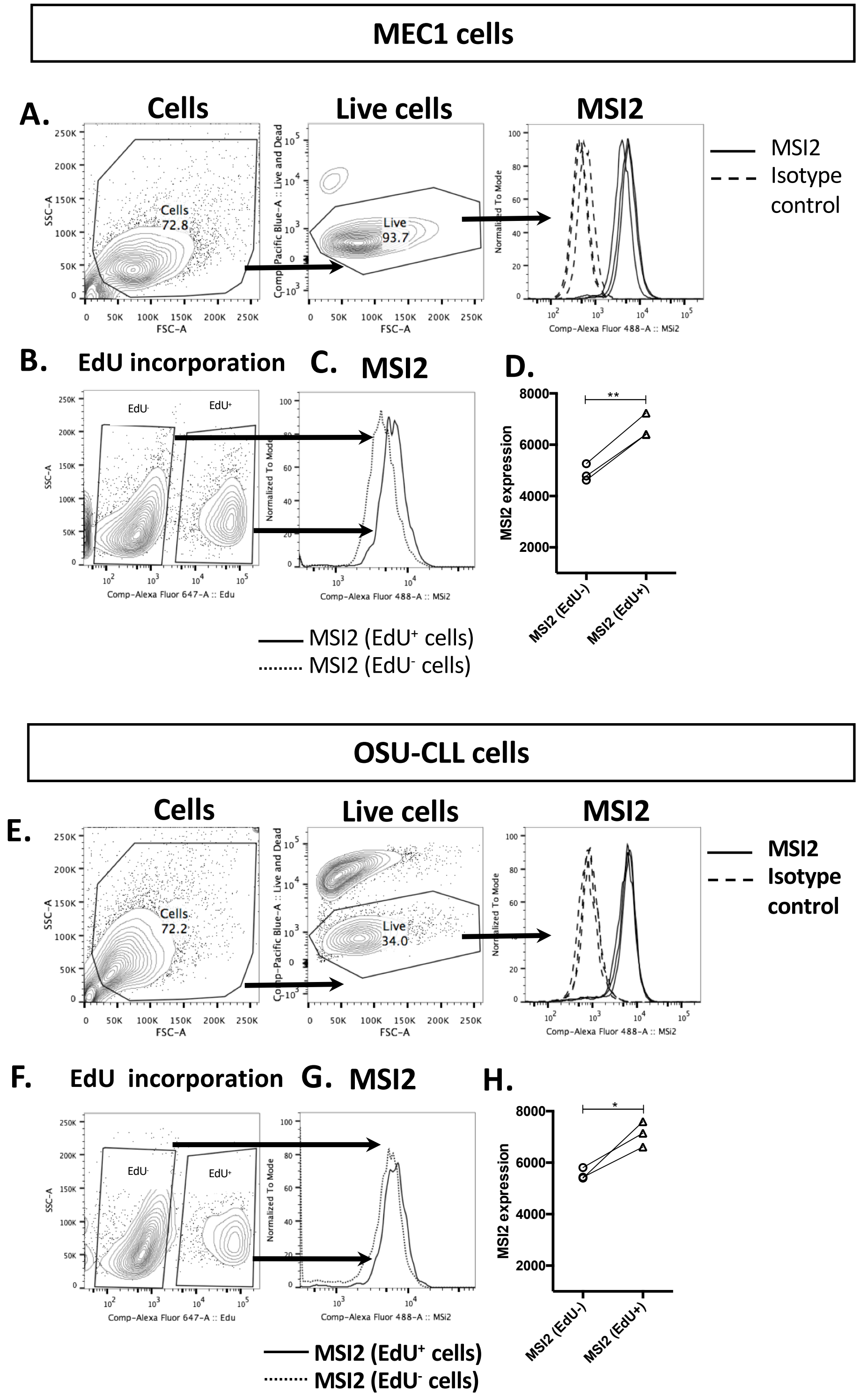
MEC1 cells
A.
Cells
Live cells
MSI2
Cells 72.8
Live 93.7
MSI2
Isotype control
B.
EdU incorporation
C.
MSI2
D.
MSI2 expression
MSI2 (EdU-)
MSI2 (EdU+)
MSI2 (EdU+ cells)
MSI2 (EdU- cells)
OSU-CLL cells
E.
Cells
Live cells
MSI2
Cells 72.2
Live 34.0
MSI2
Isotype control
F.
EdU incorporation
G.
MSI2
H.
MSI2 expression
MSI2 (EdU-)
MSI2 (EdU+)
MSI2 (EdU+ cells)
MSI2 (EdU- cells)

Figure S5

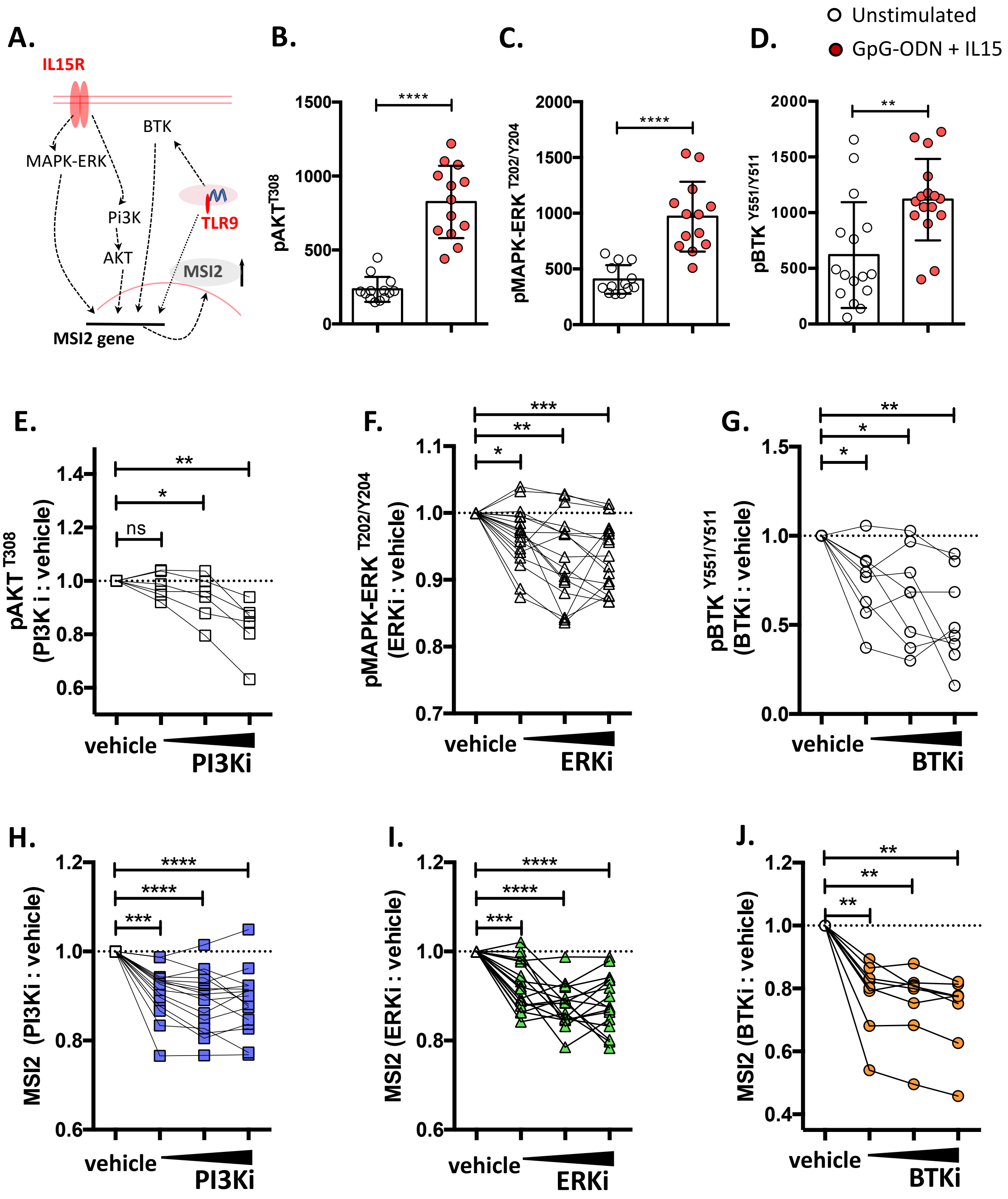
A.
IL15R
BTK
MAPK-ERK
Pi3K
AKT
TLR9
MSI2
MSI2 gene
B.
C.
D.
Unstimulated
GpG-ODN + IL15
pAKT T308
pMAPK-ERK T202/Y204
pBTK Y551/Y511
****
****
**
E.
F.
G.
pAKT T308 (PI3K i : vehicle)
pMAPK-ERK T202/Y204 (ERKi : vehicle)
pBTK Y551/Y511 (BTKi : vehicle)
ns
*
**
***
vehicle
PI3Ki
ERKi
BTKi
H.
I.
J.
MSI2 (PI3Ki : vehicle)
MSI2 (ERKi : vehicle)
MSI2 (BTKi : vehicle)
****

Figure S6

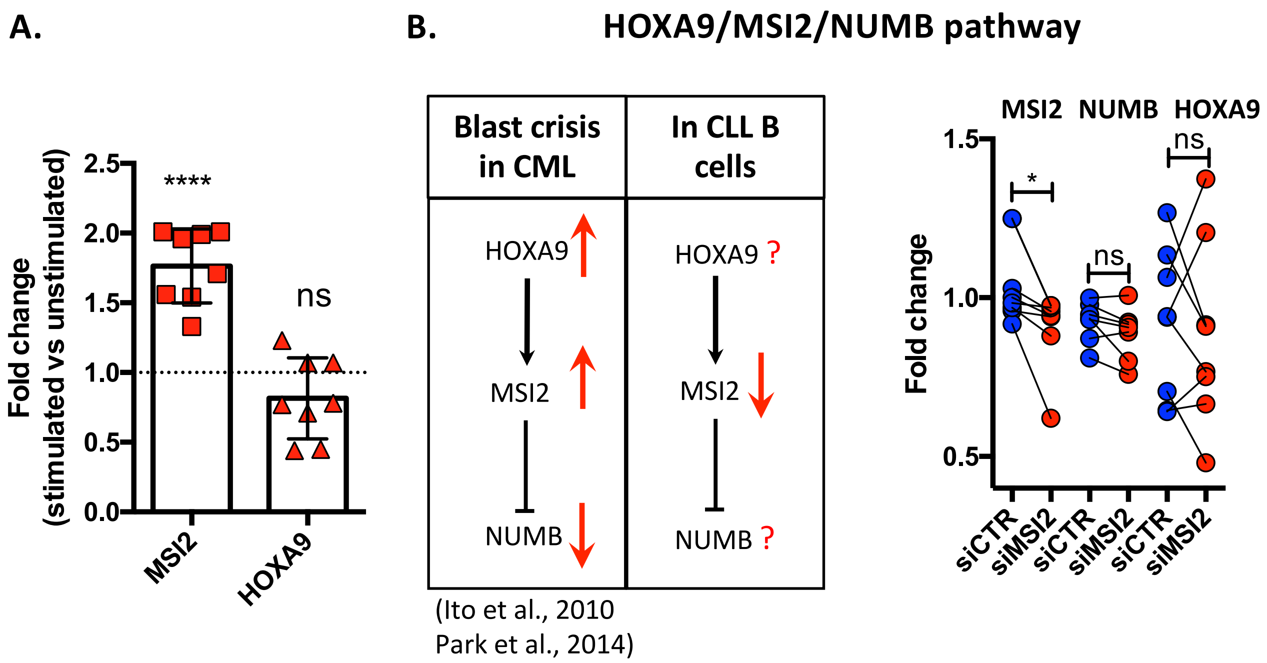
A.
B.
HOXA9/MSI2/NUMB pathway
Fold change
(stimulated vs unstimulated)
2.5
2.0
1.5
1.0
0.5
0.0
****
ns
MSI2
HOXA9
Blast crisis
in CML
In CLL B
cells
HOXA9
MSI2
NUMB
HOXA9 ?
MSI2
NUMB ?
(Ito et al., 2010
Park et al., 2014)
MSI2
NUMB
HOXA9
Fold change
1.5
1.0
0.5
*
ns
ns
siCTR
siMSI2
siCTR
siMSI2
siCTR
siMSI2

Figure S7

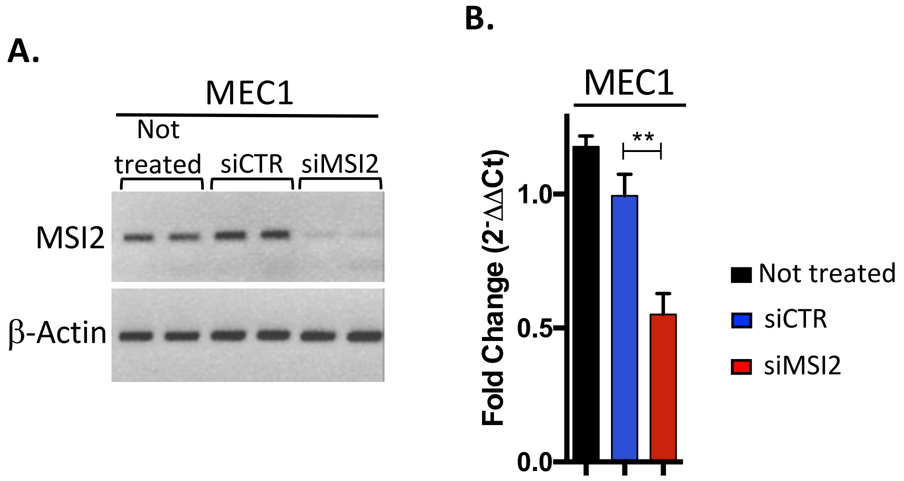
A.
MEC1
Not treated
siCTR
siMSI2
MSI2
β-Actin
B.
MEC1
Fold Change (2-ΔΔCt)
**
1.0
0.5
0.0
Not treated
siCTR
siMSI2

Figure S8

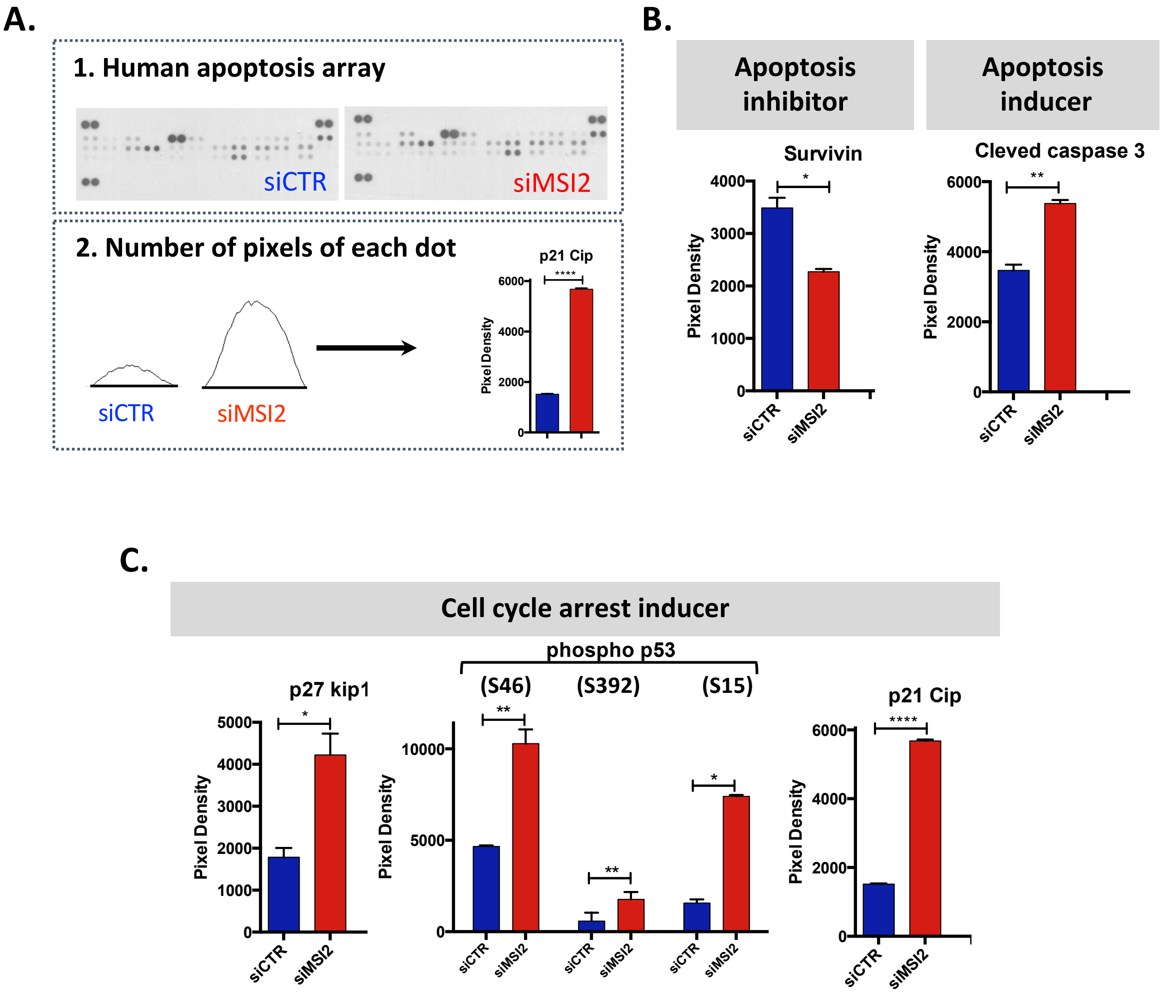

D.

	Protein	Fold Change	p value
Apoptosis inhibitors	catalase	0.3	0.00332
	PON2	0.6	0.0262
	XIAP	0.7	0.048
	Survivin	0.8	0.0135
	Hsp27	0.8	0.023
Apoptosis inducers	Cytochrome C	1.2	0.0272
	FADD	1.3	0.0466
	Procaspasa 3	1.7	0.0036
	Cleved caspasa 3	1.5	0.0051
	Death Receptor 5	1.8	0.0021
Cell cycle arrest-inducer	phospho Rad17 (S635)	1.5	0.0358
	p27-Kip1	2.4	0.025
	phospho p53 (S46)	2.2	0.0095
	phospho p53 (S392)	3.0	0.0327
	phospho p53 (S15)	4.7	0.0007
	p21-CIP1	3.7	<0.0001

Figure S9

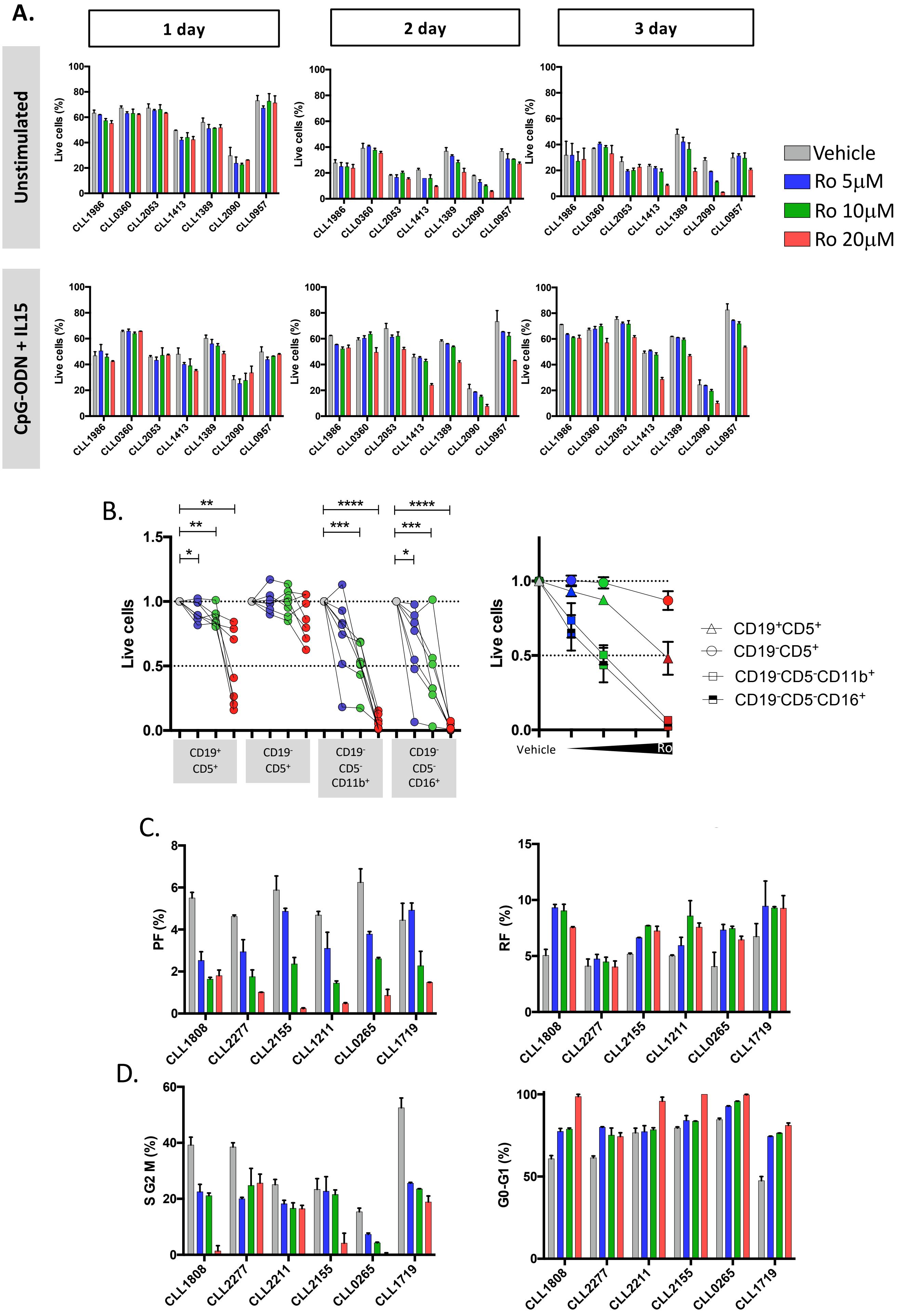
A.
1 day
2 day
3 day
Unstimulated
CpG-ODN + IL15
Live cells (%)
CLL1986
CLL0360
CLL2053
CLL1413
CLL1389
CLL2090
CLL0957
Vehicle
Ro 5μM
Ro 10μM
Ro 20μM
B.
Live cells
CD19+ CD5+
CD19- CD5+
CD19- CD5- CD11b+
CD19- CD5- CD16+
Vehicle
Ro
CD19+CD5+
CD19-CD5+
CD19-CD5-CD11b+
CD19-CD5-CD16+
C.
PF (%)
RF (%)
CLL1808
CLL2277
CLL2155
CLL1211
CLL0265
CLL1719
D.
S G2 M (%)
G0-G1 (%)
CLL2211

Figure S10

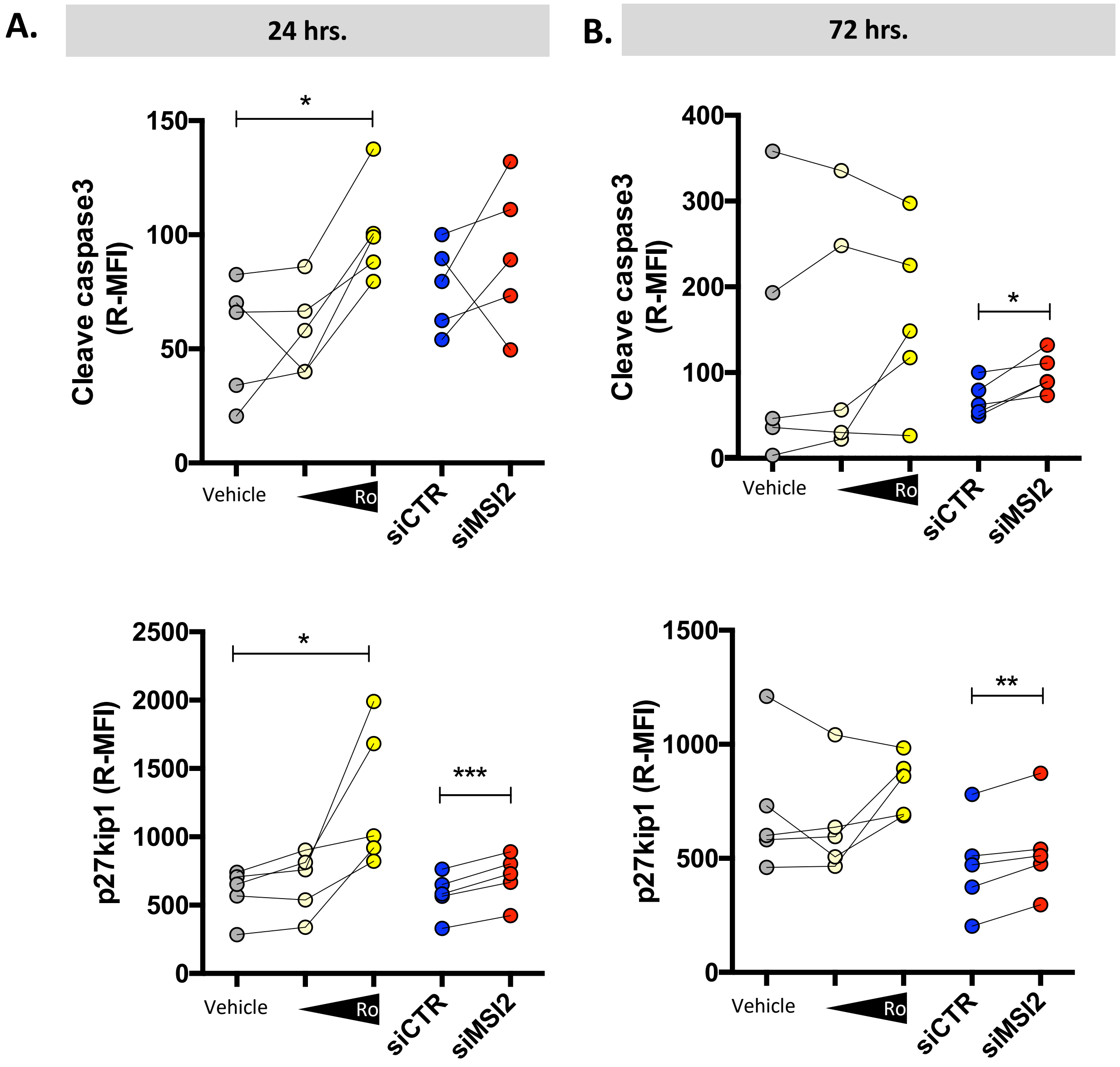
A.
24 hrs.
B.
72 hrs.
Cleave caspase3 (R-MFI)
p27kip1 (R-MFI)
Vehicle
Ro
siCTR
siMSI2
*
**
***
0
50
100
150
200
300
400
500
1000
1500
2000
2500

Figure S11

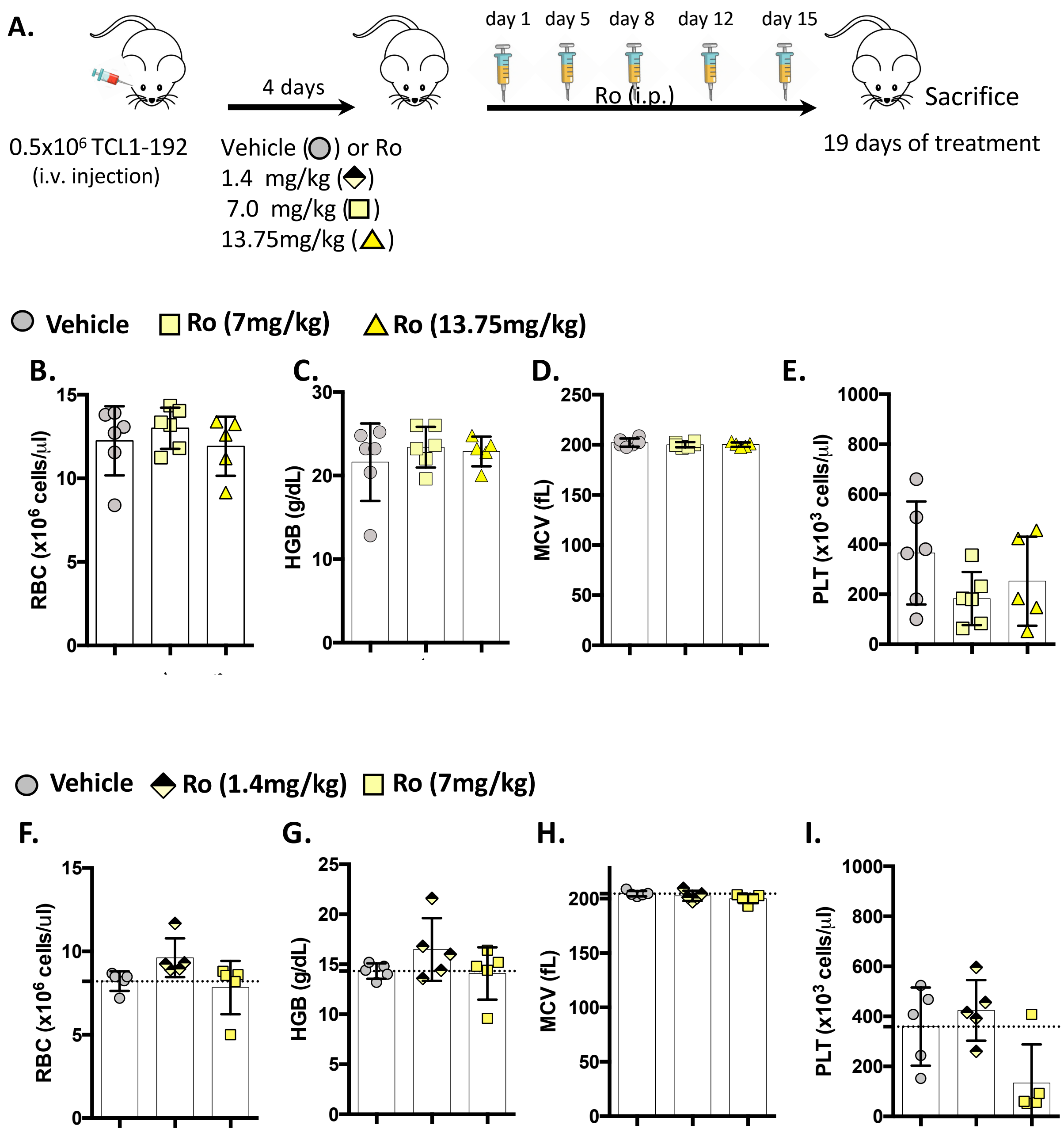
A.
day 1
day 5
day 8
day 12
day 15
4 days
Ro (i.p.)
Sacrifice
19 days of treatment
0.5x10⁶ TCL1-192
(i.v. injection)
Vehicle ( ) or Ro
1.4 mg/kg ( )
7.0 mg/kg ( )
13.75mg/kg ( )
Vehicle
Ro (7mg/kg)
Ro (13.75mg/kg)
B.
C.
D.
E.
RBC (x10⁶ cells/μl)
HGB (g/dL)
MCV (fL)
PLT (x10³ cells/μl)
Vehicle
Ro (1.4mg/kg)
Ro (7mg/kg)
F.
G.
H.
I.
RBC (x10⁶ cells/ul)
HGB (g/dL)
MCV (fL)
PLT (x10³ cells/μl)

Figure S12

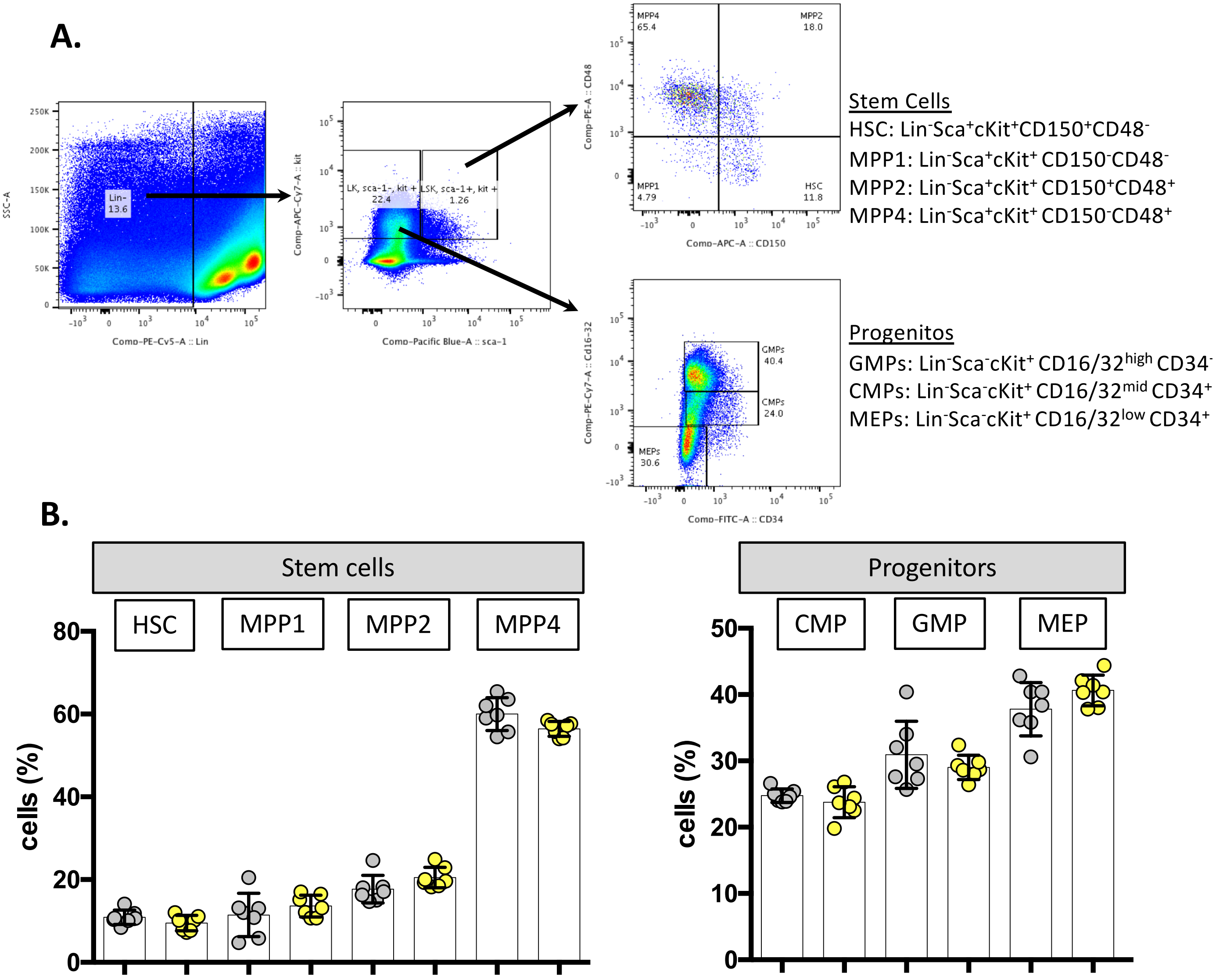
A.
SSC-A
Comp-PE-Cy5-A :: Lin
Lin-
13.6
Comp-APC-Cy7-A :: kit
Comp-Pacific Blue-A :: sca-1
LK, sca-1-, kit +
22.4
LSK, sca-1+, kit +
1.26
Comp-PE-A :: CD48
Comp-APC-A :: CD150
MPP4
65.4
MPP2
18.0
MPP1
4.79
HSC
11.8
Stem Cells
HSC: Lin-Sca+cKit+CD150+CD48-
MPP1: Lin-Sca+cKit+ CD150-CD48-
MPP2: Lin-Sca+cKit+ CD150+CD48+
MPP4: Lin-Sca+cKit+ CD150-CD48+
Comp-PE-Cy7-A :: Cd16-32
Comp-FITC-A :: CD34
GMPs
40.4
CMPs
24.0
MEPs
30.6
Progenitos
GMPs: Lin-Sca-cKit+ CD16/32high CD34-
CMPs: Lin-Sca-cKit+ CD16/32mid CD34+
MEPs: Lin-Sca-cKit+ CD16/32low CD34+
B.
Stem cells
HSC
MPP1
MPP2
MPP4
cells (%)
Progenitors
CMP
GMP
MEP

Figure S13

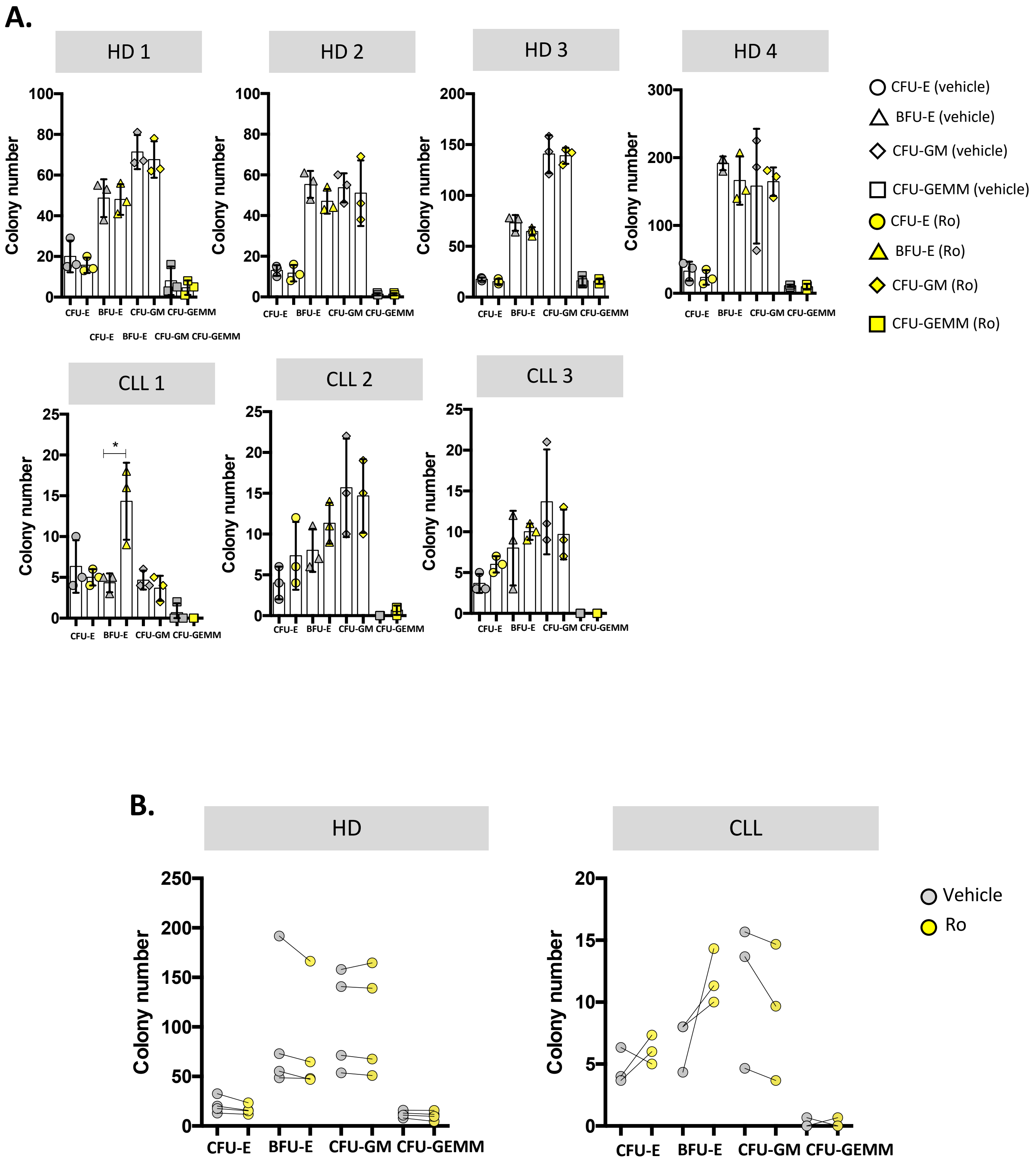
A.
HD 1
HD 2
HD 3
HD 4
CLL 1
CLL 2
CLL 3
Colony number
CFU-E
BFU-E
CFU-GM
CFU-GEMM
*
CFU-E (vehicle)
BFU-E (vehicle)
CFU-GM (vehicle)
CFU-GEMM (vehicle)
CFU-E (Ro)
BFU-E (Ro)
CFU-GM (Ro)
CFU-GEMM (Ro)
B.
HD
CLL
Vehicle
Ro

Figure S14

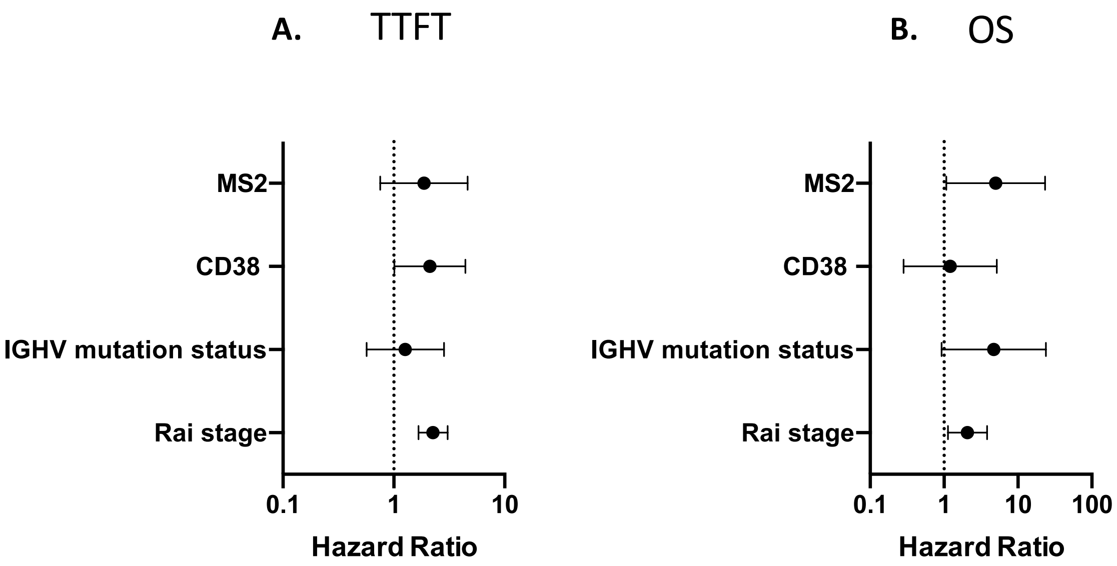
A.
TTFT
MS2
CD38
IGHV mutation status
Rai stage
0.1
1
10
Hazard Ratio
B.
OS
MS2
CD38
IGHV mutation status
Rai stage
0.1
1
10
100
Hazard Ratio

Figure S15
