# Supplementary material for: Musashi 2 influences chronic lymphocytic leukemia cell survival and growth making it a potential therapeutic target
Source: Leukemia. 2021 Jan 27;35(4):1037–52. doi: 10.1038/s41375-020-01115-y (PMC8024198; doi:10.1038/s41375-020-01115-y)
Supplement: Supplementary file 2 — Supplementary Materials and Methods [file 41375_2020_1115_MOESM2_ESM.pdf]

Supplementary Materials and Methods

Patients. Hematopoietic stem and progenitor cells were collected from bones removed at surgery from patients with degenerative joint disease after obtaining informed consent. For all the experiments using patient samples, the investigator was blinded to the group allocation during the experiment. IRB # 08-202A

Mice. The sample size choice of 5 (2 month old female) mice per treatment group, total 3 experiments, was based on feasibility, and the analysis was performed as a two-sample t-test (alpha=0.05). The investigator was blinded to the treatments and analysis. IACUC # 2010-004

Cell lines. OSU-CLL and MEC1 were kindly provided by Drs. John C. Byrd (Ohio State University, Columbus, Ohio, USA) and Emili Montserrat (Hospital Clinic, University of Barcelona, Spain) and both cell lines were tested for mycoplasma contamination.

Measurements of surface and intracellular antigens by flow cytometry. Live cells were identified using LIVE/DEAD Fixable Stains for flow cytometry (LIVE/DEAD™ Fixable Violet Dead Cell Stain Kit or Far Red Dead Cell Stain Kit, Life Technology). For surface membrane immunofluorescence, cells (2×10^5) in FACS buffer (PBS + 10% bovine serum albumin + 1% sodium azide) were incubated with primary antibody for 20 min at 4°C, followed by fixation with 0.1% formaldehyde in PBS. For intracellular detection of MSI2, HOXA9, Ki-67, p27Kip1, p21Cip1, cleaved caspase 3, survivin, phosphor, AKT, MAP-ERK, BTK, p53, after surface membrane staining with anti-CD19, -CD5 and -CXCR4,

cells were fixed and permeabilized (Cytofix/Cytoperm, BD Biosciences) and incubated with murine antibody. The expression level of MSI2 was defined by the relative mean fluorescence intensity (MFI-R), which is the mean fluorescence intensity (MFI) of CLL cells stained for MSI2 minus the MFI of the same cells stained with isotype control. Data were acquired with a BD LSR Fortessa flow cytometer and analyzed by FlowJo X version. Within each CLL clone, relative expression in terms of percent positive cells and mean fluorescent intensity of each surface or intracellular marker was determined and compared

Culture conditions for CLL B-cell stimulation. Cultures were established in 96 round bottom well plates at 3x105 cells per 150 μl volume with triplicates for each culture condition. Recombinant CD40L (MEGACD40L, Enzo) and IL-4 (PeproTech) were use at 0.1 μg/mL and 20 ng/mL, respectively. For CpG-ODN+IL-15 stimulation, recombinant human IL- 15 (PeproTech) and CpG DNA TLR-9 ligand (ODN-2006; Invivogen) were added at final culture concentrations of 15 ng/ml and 1.5 μM, respectively.

Cell division and cell cycle phase were evaluated using cell trace (Cell trace Far red cell proliferation kit, Life Technology) and FxCycle (FxCycle™ Violet Stain, Thermo Fisher Scientific), respectively. To determine the level of EdU incorporation, Click-iT™ EdU Alexa Fluor™ 647 Imaging Kit (ThermoFisher Scientific) was used.

Colony forming unit assay. Frozen bone marrow cells of healthy donors and CLL patients were thawed in warm RPMI containing 20% FBS followed by Ficoll density centrifugation to remove dead cells. The enrichment of $CD34^+$ cells were performed by

MACS Separation using CD34 MicroBead Kit (Milteyi Biotec) and LS columns (Milteyi Biotec). After purification, $CD34^+$ cells were cultured overnight in IMDM medium (HyClone) containing 20% BIT 9500 (Stem Cell Technologies), 100ng/ml SCF, 20ng/ml IL-6, 100ng/ml TPO and 10ng/ml FLT-3 ligand (PeproTech). For colony forming unit (CFU) assays, the $CD34^+$ cells from healthy donors (3000 cells) and CLL patients (1000 cells) were plated (in triplicate) in methylcellulose (MethoCultTM H4434 Classic, Stem Cell Technologies). CFU colonies corresponding to erythroid progenitor cells (BFU-E), granulocyte-macrophage progenitor cells (CFU-GM, CFU-G and CFU-M), and multipotent granulocyte, erythroid, macrophage and megakaryocyte progenitor cells (CFU-GEMM) were scored 14 days after seeding.
