# Supplementary material for: Musashi 2 influences chronic lymphocytic leukemia cell survival and growth making it a potential therapeutic target
Source: Leukemia. 2021 Jan 27;35(4):1037–52. doi: 10.1038/s41375-020-01115-y (PMC8024198; doi:10.1038/s41375-020-01115-y)
Supplement: Supplementary file 3 — Table S1A [file 41375_2020_1115_MOESM3_ESM.pdf]

Table S1A: Untreated CLL patients from whom samples were used to determine MSI2 protein levels after *in vitro* stimulation (Fig. 2; effect on cell survival) downregulated/inhibited its function in CLL cells (Figs. 3-4 and 6, respectively) and the relationship of MSI2 protein levels with clinical course (TTFT and OS, Fig. 5).

CLL number	Gender	Rai stage	IGHV mutation status	Diagnose date	Sample date	Treatment date	Theraphy received	Death Date	TTFT (years)	OS (years)	MSI2 protein levels (MFI)	In vitro stimulation of CLL cells (Fig. 2)	MSI2 downregulation using siRNA (Figs. 3 and 4)	Outcome, TTFT and OS (Fig. 5)	Blocking of MSI2 function by molecular inhibition (Fig.6)
360	M	1	UM												✓
373	F	2	M									✓			
545	F	3	M									✓	✓		
614	F	0	UM										✓		
635	M	1	UM									✓			
699	M	0	M									✓			
957	F	0	UM												✓
994	F	1	M									✓			
1019	M	0	M									✓			
1024	F	0	M										✓		✓
1082	M	1	M	5/1/07	4/10/10	4/26/11	FCR		3.0	8.6	740			✓	
1093	M	0	UM									✓			
1159	M	0	M	1/1/01	1/11/08				7.5	13.4	1053			✓	
1235	M	2	UM										✓		
1320	F	2	M	1/1/04	8/17/11	1/15/04	Chlorambucil	2/17/12	0.0	8.1	953			✓	
1324	M	2	UM	11/1/08	2/12/09			9/1/11	0.6	2.8	1078			✓	
1326	F	3	UM	1/1/04	2/12/08	12/1/08	R		4.9	10.4	1038			✓	
1333	F	1	UM	1/1/99	3/5/09	12/1/09	FR		10.9	15.4	1349			✓	
1334	F	0	M	6/1/08	3/10/09				7.5	7.5	1267			✓	
1340	M	2	UM	8/9/07	3/25/09				1.6	6.8	732			✓	
1341	M	0	M	12/1/08	3/26/09				9.2	9.2	997			✓	
1346	F	0	M	4/7/09	1/27/12				7.3	7.4	1090			✓	
1352	F	1	M	4/1/09	5/6/09			4/2/11	0.1	2.0	953			✓	
1358	M	1	M	9/11/06	5/15/09				2.7	7.7	1016			✓	
1380	M	1	M	4/1/08	5/25/11				3.2	6.2	1266			✓	
1383	F	0	M	7/1/98	11/16/10				12.4	15.9	1059			✓	
1384	M	2	UM	7/1/05	7/9/09	3/8/10	FCR	9/18/11	4.7	6.2	1702			✓	
1386	M	0	UM	11/1/08	12/22/10				2.1	5.6	1061			✓	
1388	M	1	M	1/1/01	7/20/09				8.5	13.4	1059			✓	
1389	F	0	M	1/11/11				12/12/15					✓		✓
1402	M	0	M	7/1/06	9/24/10			8/18/14	7.8	7.9	799			✓	
1413	F	1	M												✓
1415	M	1	M										✓		
1455	M	1	UM									✓			
1493	F	1	M									✓			
1496	M	1	UM	4/15/10	5/26/10				0.1	4.1	1189			✓	
1498	M	2	M					7/18/17				✓			
1552	M	0	M					7/11/16				✓			
1572	M	1	UM	1/11/11	3/1/11	4/22/11	RCD	12/12/15	0.3	4.9	1248			✓	

1618	M	2	UM	1/1/09	7/12/11	2/6/12	BR		3.1	8.6	1177			✓	
1636	F	0	M					7/25/17				✓			
1719	F	1	UM					6/28/17				✓			
1737	M	4	M	6/1/04	2/7/14			8/18/14	8.0	10.2	805			✓	
1746	M	2	UM	12/1/08	11/10/14				5.9	5.9	1782			✓	
1747	F	0	M	8/1/11	7/12/12	5/1/13	BR		1.8	6.1	884			✓	
1843	M	0	M	11/27/13				5/30/17				✓			
1851	M	1	UM	10/1/04	5/23/13			10/22/14	9.5	10.1	1128			✓	
1853	M	0	M	1/1/98	6/7/13				15.4	16.4	773			✓	
1860	M	0	UM	1/1/11				3/15/16					✓		
1863	M	0	M	5/10/05	7/29/15	4/19/16	FCR		10.9	12.2	722			✓	
1873	F	0	M					2/8/18				✓			
1875	M	4	UM					7/1/14				✓			
1883	M	3	UM	5/9/06	10/22/13				7.5	10.2	1209			✓	
1885	M	3	UM	6/1/11	11/15/13	2/1/14	FCR		2.7	6.1	804	✓		✓	
1889	F	0	UM	5/26/10	2/10/14	11/26/13	Ibrutinib		3.5	6.8	751			✓	
1893	M	1	M	9/1/13	9/15/14	8/12/15	RI		1.9	3.9	1482			✓	
1893	M	2	UM	1/1/10				11/11/14				✓			
1895	M	NA	UM	6/1/14				8/7/17				✓			
1904	M	3	UM	12/1/13	9/22/14	2/1/14	Chlorambucil		0.2	3.6	1204			✓	
1906	M	1	UM	6/1/09	1/24/14			4/5/17	7.8	7.8	862			✓	
1910	F	1	UM	1/1/14	2/7/14	3/1/14	BR		0.2	3.3	1372	✓		✓	
1911	M	2	UM	10/1/13	2/7/14	7/9/15	Chlorambucil		1.8	4.5	1241			✓	
1913	M	2	UM	11/27/13	2/21/14				3.5	3.5	1015			✓	
1913	F	0	M					4/20/17				✓	✓		
1924	F	0	M	1/1/13	6/3/15				5.1	5.1	1087			✓	
1924	M	1	UM									✓	✓		
1925	M	1	UM	12/1/14	3/25/15	12/1/16	FCR		2.0	3.4	1011			✓	
1926	M	2	M	1/1/11	1/22/14	2/1/14	Ibrutinib		3.1	5.2	786			✓	
1932	F	1	M									✓			
1936	M	NA	UM									✓	✓		
1953	M	2	UM	1/1/14	3/6/15	11/9/15	FCR	3/17/16	1.9	2.2	989			✓	
1955	F	1	UM			1/1/12						✓	✓		
1958	M	2	UM	8/1/07	12/17/14	9/1/07	FR		0.1	10.5	1109			✓	
1961	M	2	UM	7/2/12	7/2/14				2.0	2.0	814			✓	
1961	M	2	UM									✓	✓		
1970	M	1	M	1/1/09	7/18/14				8.3	8.5	1366	✓		✓	
1972	F	2	UM	7/23/14	7/23/14						779			✓	
1978	M	1	M	6/1/14	8/6/14				3.1	3.1	1008			✓	
1982	F	0	M									✓	✓		
1985	F	4	M									✓	✓		
1986	F	0	UM												✓
1988	F	3	UM	11/1/12	10/1/14	10/1/14	FCR		1.9	3.3	1099			✓	
1990	F	0	M	1/1/10	11/11/14				4.9	4.9	1038			✓	
1991	M	1	M	6/1/14	10/17/14	11/13/14	Ibrutinib		0.5	3.2	842			✓	
2002	M	0	M	1/1/10	1/15/15				5.3	5.3	767	✓		✓	
2003	F	0	M									✓			
2010	M	0	UM	2/1/13	2/24/15				2.1	2.1	1026	✓		✓	

2011	M	1	M	1/1/91	2/25/15	1/1/08	Rituximab	1/25/16	17.0	25.1	1008	✓		✓	
2012	M	NA	UM									✓			
2018	M	0	M	1/1/09	4/8/15				8.3	8.3	559	✓	✓	✓	
2019	M	3	UM	1/1/11	4/9/15	10/16/14	Chlorambucil	6/23/15	3.8	4.5	1330	✓	✓	✓	
2027	M	3	UM	1/1/07	5/21/15	1/1/12	Bendamustine		5.0	10.3	787			✓	
2049	F	NA	M										✓		
2053	M	NA	UM												✓
2059	M	1	UM									✓	✓		
2067	M	NA	UM									✓	✓		
2081	M	NA	UM										✓		
2085	F	NA	UM									✓			
2090	M	1	M												✓
2094	F	2	M										✓		
2101	F	1	UM									✓			
2122	F	1	UM									✓			
2131	M	0	UM									✓	✓		
2156	M	3	UM									✓			

Patients were treated with: FCR (Fludarabine, Cyclophosphamide, Rituximab), Chlorambucil , Rituximab, FR (Fludarabine, Rituximab), Rituximab Cyclophosphamie Decadron (RCD), Bendamustine Rituximab (BR), Ibrutinib, Rituximab Ibrutinib (RI)

Samples were collected from patients cared for at Northwell Health.

M: mutated IGVH gene. **UM:** unmutated IGVH gene

TTFT: Time to first treatment. **OS:** Overall survival
