# Supplementary material for: Musashi 2 influences chronic lymphocytic leukemia cell survival and growth making it a potential therapeutic target
Source: Leukemia. 2021 Jan 27;35(4):1037–52. doi: 10.1038/s41375-020-01115-y (PMC8024198; doi:10.1038/s41375-020-01115-y)
Supplement: Supplementary file 4 — Table S1B [file 41375_2020_1115_MOESM4_ESM.pdf]

Table S1B: Untreated CLL patients from whom samples were used to determine the relationship of MSI2 mRNA levels with clinical course (TTFT and OS).

CLL no.	Gender	Age	Rai stage	IGHV mutation status	Diagnose date (Dx)	Sample date (Sx)	Treatment date	Theraphy received	Death Date	CD38 (%)	Cytogenetics	TTFT (-->Dx, years)	OS (-->Dx, years)	TTFT (-->Sx, years)	OS (-->Sx, years)	relative MSI2 (dCT)
CLL#1	F	65	3	UM	6/24/04	04/16/08	7/18/08	PCR	12/26/11	93	normal	4.07	7.51	0.25	3.70	1.64
CLL#2	M	62	0	M	3/20/12	3/20/12				21	del 13q14	0.73	0.73	0.73	0.73	2.56
CLL#3	M	73	4	UM	9/1/03	01/22/09	3/24/10	PCR	6/16/10	14	del 11q (70%)	6.56	6.79	1.17	1.40	2.82
CLL#4	M	73	1	UM	10/28/03	08/07/08	8/30/08	R-CHOP	1/13/10	41	del13q (15.6%)	4.84	6.22	0.06	1.44	2.82
CLL#5	F	54	0	M	2/22/08	1/6/12				13	normal	4.54	4.54	0.66	0.66	2.83
CLL#6	M	78	1	UM	5/6/11	6/19/12				88	normal	1.66	1.66	0.53	0.53	2.88
CLL#7	F	63	1	M	6/26/99	6/13/12				5	normal	13.47	13.47	0.50	0.50	2.98
CLL#8	F	65	0	M	10/26/09	2/7/12				40	normal	3.20	3.20	0.92	0.92	3.00
CLL#9	M	73	4	UM	1/1/04	08/07/08	1/1/09	PCR	1/8/09	75	NORMAL	5.01	5.02	0.40	0.42	3.38
CLL#10	M	54	1	UM	5/8/06	9/11/09	11/2/11	PCR, CAR Tcells		6	normal	5.49	6.70	2.14	3.35	3.45
CLL#11	F	68	0	M	5/11/09	3/20/12				16	loss atm, del 13q14	3.59	3.59	0.73	0.73	3.46
CLL#12	M	67	0	M	1/1/00	11/13/08	10/4/09	Cisplatin and (	10/29/10	27	2 clones: trisomy 12 (50%) and del 13q (6%)	9.76	10.83	0.89	1.90	3.47
CLL#13	F	75	3	M	2/28/08	12/16/08	12/16/08	R-CVP	8/4/10	47	del 11q(88%), del 17p (88%)	0.80	2.43	0.00	1.63	3.49
CLL#14	M	55	2	UM	3/29/07	1/10/12	2/16/12	PCR		3	del 11q	4.89	5.85	0.10	1.06	3.52
CLL#15	F	66	0	M	8/18/08	11/20/08				69	normal	3.43	3.43	3.18	3.18	3.56
CLL#16	M	45	0	UM	10/24/07	04/20/08	10/4/12	BR		6	del 13q (85%)	4.95	4.49	4.46	4.00	3.60
CLL#17	M	59	3	M	12/10/07	03/21/08	6/12/08	PCR		41	del13q (13.7%)	0.51	4.94	0.23	4.66	3.61
CLL#18	M	64	0	M	11/18/04	11/13/08				17	normal	8.13	8.13	4.14	4.14	3.63
CLL#19	M	48	1	M	7/1/08	05/01/09				26	del13q14.3 (69%)	1.01	1.01	0.17	0.17	3.63
CLL#20	M	59	4	UM	1/13/10	10/04/11	10/4/11	PCR		70	normal 9/27/11	1.72	2.84	0.00	1.12	3.68
CLL#21	M	72	1	M	3/10/05	6/20/12				6	del 13q	7.85	7.85	0.56	0.56	3.68
CLL#22	F	60	0	M	1/6/06	10/16/09				14	del 13q 91.2% 1/6/06	6.90	6.90	3.13	3.13	3.87
CLL#23	M	46	1	UM	8/14/06	07/31/08	7/9/09	PCR		69	del13q (96.6%)	2.90	6.41	0.94	4.44	4.00
CLL#24	M	52	0	M	2/7/08	05/23/08				23	normal	0.29	0.29	0.00	0.00	4.07
CLL#25	F	76	1	UM	5/21/04	04/16/08				64	trisomy 12 (75%)	8.48	8.48	4.57	4.57	4.16
CLL#26	F	62	0	UM	3/19/08	5/23/12				6	del 13q	4.69	4.69	0.51	0.51	4.16
CLL#27	M	52	1	M	8/10/09	10/23/09				15	del 13q14 in 37.4% in 10/2009	3.26	3.26	3.06	3.06	4.24
CLL#28	M	77	0	M	1/1/07	11/07/08				13	del 11q (81%) del 13q (83.6%)	5.92	5.92	4.07	4.07	4.28
CLL#29	M	84	0	M	1/1/04	02/25/09			8/8/11	12	del 13q	7.26	7.61	2.11	2.11	4.29
CLL#30	M	76	0	M	9/1/00	11/18/09				25	NR	12.21	12.21	2.99	2.99	4.30
CLL#31	F	65	0	UM	7/1/00	04/02/08	12/1/11		2/3/12	21	not tested	11.42	11.60	3.67	4.84	4.33
CLL#32	M	65	0	M	5/23/05	12/11/08				19	del 13q (32% 6/23/05)	5.22	5.22	1.66	1.66	4.36
CLL#33	F	70	1	M	4/8/08	12/11/08				8	del 13q (94%)	4.65	4.65	3.97	3.97	4.37
CLL#34	M	58	2	M	6/25/02	09/25/08	10/12/11	PCR	8/5/12	9	del 13q (65%)	9.30	10.12	3.05	3.86	4.39
CLL#35	M	52	0	M	9/27/11	6/19/12				17	del 13q	1.44	1.44	0.71	0.71	4.40
CLL#36	F	53	2	UM	3/24/04	11/14/08	5/2/09	R-CHOP ALLO		63	TRISOMY 12 (65%)	5.11	8.65	0.46	4.01	4.41
CLL#37	M	70	0	M	1/18/10	07/16/10				8	del 13q14 9% 7/16/10	2.87	2.87	2.38	2.38	4.42
CLL#38	M	68	0	M	6/3/09	09/30/11				7	del 13q14 46% 6/18/10	2.33	2.33	0.00	0.00	4.43
CLL#39	F	84	2	M	11/30/06	09/26/12	9/27/12	R-CVP		76	del 13q ; partial deletion of IgH	5.83	6.31	0.00	0.48	4.52
CLL#40	M	65	1	UM	12/17/09	04/02/10	1/18/12	BR		100	trisomy 12 90.4% 1/26/10	2.09	2.99	1.80	2.70	4.54
CLL#41	M	73	1	UM	11/20/07	06/06/08	12/13/11	PCR		68	normal 12/28/07	4.07	4.99	3.52	4.45	4.54
CLL#42	M	59	0	UM	1/29/08	05/07/08				71	del 11q (94%)	4.82	4.82	4.55	4.55	4.56
CLL#43	F	50	0	M	6/27/07	10/30/09				34	del 13q in 31% 2007	3.57	3.57	1.23	1.23	4.59
CLL#44	M	58	0	M	4/23/99	05/09/07				ND	ND	13.04	13.04	4.99	4.99	4.63
CLL#45	F	73	0	UM	9/1/08	06/12/09				63	normal	0.83	0.83	0.05	0.05	4.65
CLL#46	F	76	1	M	10/1/04	10/25/07	1/7/10	LEN		11	normal	5.27	8.32	2.21	5.25	4.67
CLL#47	M	48	1	M	3/1/12	6/12/12				17	trisomy 12, del 14q	0.80	0.80	0.52	0.52	4.68
CLL#48	M	68	0	M	10/3/08	03/20/09				6	del 13q (10%)	4.14	4.14	3.68	3.68	4.70
CLL#49	M	61	1	UM	9/5/08	3/13/12				16	del 13q14	4.02	4.02	0.50	0.50	4.73
CLL#50	M	55	0	UM	12/2/11	3/6/12				12	normal	0.94	0.94	0.68	0.68	4.73
CLL#51	F	46	0	M	9/29/09	5/22/12				13	normal	3.17	3.17	0.52	0.52	4.75
CLL#52	M	41	0	UM	4/13/10	09/29/11				ND	loss atm 97% and del 13q14 95% 12/21/10	2.42	2.42	0.96	0.96	4.77
CLL#53	F	70	1	M	1/1/03	09/29/11				23	trisomy 12 (96%) 6/28/11	10.01	10.01	1.27	1.27	4.83
CLL#54	M	71	2	UM	3/1/06	09/28/07				27	del 11q (92%) del 13q (95%)	6.79	6.79	5.21	5.21	4.87
CLL#55	M	71	0	M	5/26/09	11/06/09				28	del 13q14 in 40.8% 11/6/09	2.06	2.06	1.61	1.61	4.87
CLL#56	F	64	2	UM	11/20/97	09/29/11	9/10/12	LEN		70	normal 2/25/2011	14.82	15.19	0.95	1.32	4.94
CLL#57	M	62	0	M	4/1/07	06/11/09				56	del p53 94% 6/7/07	5.60	5.60	3.41	3.41	4.96
CLL#58	M	71	1	M	4/1/04	11/29/07	5/14/11		7/3/11	17	del 13q (20%)	7.12	7.26	3.46	3.59	5.01
CLL#59	M	71	1	M	1/1/95	10/12/07	9/1/08	PCR, RCVP, RTX		77	normal	13.68	18.10	0.89	5.31	5.04
CLL#60	M	54	1	M	5/1/03	07/31/08				25	normal	9.59	9.59	4.33	4.33	5.07

CLL#61	F	76	1	UM	4/1/05	04/09/08				96	normal	3.02	3.02	0.00	0.00	5.08
CLL#62	M	52	2	UM	2/1/07	09/27/11				83	loss of p53 in 72.6% 4/2007	5.85	5.85	1.19	1.19	5.08
CLL#63	M	55	2	UM	3/15/05	05/22/07	2/1/12	PCR		62	normal	6.89	7.79	4.70	5.61	5.09
CLL#64	F	70	2	UM	9/28/07	11/14/07			10/1/11	37	del 13q (82%)	0.67	4.01	0.54	3.88	5.09
CLL#65	M	85	0	M	8/24/06	6/19/12				16	del 13q14.3	6.32	6.32	0.50	0.50	5.11
CLL#66	M	64	0	M	10/1/04	06/22/07				ND	del13q	2.72	2.72	0.00	0.00	5.14
CLL#67	F	56	0	UM	11/1/05	10/30/08				71	normal	7.22	7.22	4.22	4.22	5.15
CLL#68	F	51	0	M	5/23/11	09/29/11				10	normal 6/24/2011	1.30	1.30	0.94	0.94	5.28
CLL#69	F	81	0	M	6/19/03	02/12/10	9/21/12	LEN		17	normal 7/15/2003	9.27	9.61	2.61	2.95	5.30
CLL#70	M	62	4	M	1/1/89	10/16/08	10/22/08	PCR		13	del 13q (75%)	19.82	24.09	0.02	4.29	5.33
CLL#71	F	64	0	M	12/27/05	09/29/11				23	normal 2/9/06	6.76	6.76	1.00	1.00	5.33
CLL#72	M	87	0	M	11/1/04	06/22/07				11	del 13q (40% 2/22/05)	7.94	7.94	5.30	5.30	5.34
CLL#73	F	69	0	M	1/1/00	05/10/07				0.21	normal	12.92	12.92	5.56	5.56	5.36
CLL#74	M	66	1	UM	7/1/04	11/20/08	11/27/08	PCR		94	del 11q (79.6%), TRISOMY 12 (68.4), t (10;	4.41	8.43	0.02	4.04	5.42
CLL#75	F	47	0	M	7/1/05	05/11/07				16	NORMAL	7.25	7.25	5.39	5.39	5.45
CLL#76	F	81	1	M	4/22/04	11/11/11	2/17/12	CVP		9	normal 10/22/10	7.83	8.63	0.27	1.07	5.46
CLL#77	M	64	0	M	1/1/07	04/16/10				8	normal 4/16/10	5.40	5.40	2.11	2.11	5.51
CLL#78	M	62	1	UM	8/21/06	10/12/07	3/1/09	FCR, R-CHOP		36	TRISOMY 12(2%)	2.53	6.34	1.39	5.20	5.55
CLL#79	F	57	0	M	9/1/00	02/12/09	1/19/11	PCR		8	del 13q (homozygous 70%, heterozygous 15%	10.39	12.31	1.93	3.85	5.63
CLL#80	F	80	1	M	1/1/02	07/09/10				11	del 13q in 95% del 13q14 in 75% 9/29/2006	10.92	10.92	2.40	2.40	5.69
CLL#81	M	60	1	M	2/28/07	05/10/07			11/9/12	10	del13q14 (73%)	5.61	5.70	5.42	5.42	5.71
CLL#82	F	61	0	UM	6/2/05	12/16/08				80	Trisomy 12 and del13q (5.6% 6/30/05)	7.50	7.50	3.96	3.96	5.73
CLL#83	F	79	0	UM	5/25/07	06/27/07				13	del13q (78% 10/17/07)	3.41	3.41	3.32	3.32	5.77
CLL#84	M	50	0	M	5/18/06	05/19/06				12	normal	5.87	5.87	5.87	5.87	5.78
CLL#85	M	55	0	UM	3/1/08	11/20/08	11/1/09	died prior to T	11/25/09	75	del 11q (42%), del 13q (83.6%)	1.67	1.74	0.95	1.01	5.90
CLL#86	M	54	1	M	3/23/10	6/19/12				22	del 13q	2.24	2.24	0.00	0.00	5.90
CLL#87	M	32	0	M	8/20/07	11/02/07				14	normal	3.23	3.23	3.02	3.02	5.92
CLL#88	F	76	0	UM	6/20/07	06/20/07	3/30/10	PCR		89	del 11q (40%) trisomy 12 (70%) del 13q (14%	2.78	5.47	2.78	5.47	5.93
CLL#89	M	58	3	M	12/31/86	6/15/12				29	normal	26.14	26.14	0.67	0.67	5.93
CLL#90	M	62	0	M	2/23/07	10/30/09				13	normal 5/12/06	5.28	5.28	2.60	2.60	5.96
CLL#91	F	58	0	M	8/3/05	04/16/10				15	normal 12/15/05	7.31	7.31	2.61	2.61	5.97
CLL#92	M	64	3	UM	8/17/07	4/18/12	4/26/12	PC		12	trisomy 12	4.70	5.44	0.02	0.77	6.06
CLL#93	M	38	0	M	8/2/06	05/18/07				7	NORMAL	6.52	6.52	5.73	5.73	6.07
CLL#94	F	62	3	UM	10/31/09	07/13/10	7/23/10	R-CVP	9/8/11	79	del 13q14 98% and loss of p53 7/13/10	0.73	1.85	0.03	1.14	6.08
CLL#95	M	69	0	M	7/20/99	08/20/08				18	NR	13.38	13.38	4.28	4.28	6.08
CLL#96	M	67	0	M	12/1/06	12/11/08				16	del 13q (11%)	5.96	5.96	3.93	3.93	6.13
CLL#97	F	52	1	M	1/28/11	09/27/11				20	normal 4/7/2011	1.39	1.39	0.73	0.73	6.15
CLL#98	M	59	1	UM	6/19/07	06/19/07	1/1/10	PCR		13	del 13q (11%)	2.54	5.41	2.54	5.41	6.15
CLL#99	M	66	3	M	5/11/05	12/11/08	4/1/10	PCR		12	normal karyotype FISH: trisomy 12 in 9.2 %	4.89	7.34	1.30	3.75	6.19
CLL#100	M	49	0	M	8/19/04	12/18/08				23	normal	7.76	7.76	3.43	3.43	6.21
CLL#101	M	68	0	M	12/19/05	12/11/08				20	normal	6.99	6.99	4.01	4.01	6.29
CLL#102	M	69	0	M	7/1/98	10/26/07			1/27/11	ND	normal	12.50	12.58	3.18	3.26	6.31
CLL#103	M	74	0	M	1/1/05	06/07/07				5	del 13q (82%)	7.81	7.81	5.38	5.38	6.34
CLL#104	F	71	0	M	9/1/01	06/08/07				14	ND	10.68	10.68	4.91	4.91	6.47
CLL#105	M	64	1	UM	7/26/05	04/16/08			10/4/10	27	normal	3.41	5.19	0.68	2.47	6.90
CLL#106	M	69	0	M	2/14/00	06/27/07				36	ND	12.67	12.67	5.30	5.30	7.01
CLL#107	F	57	0	M	1/1/06	10/13/07				14	del 13q (68%)	6.32	6.32	4.54	4.54	7.03
CLL#108	M	54	4	M	5/1/04	07/30/08	2/2/09	FCR		3	del 13q	4.76	5.67	0.51	1.42	7.10
CLL#109	M	78	0	M	3/1/95	03/05/08				ND	not tested	17.55	17.55	4.53	4.53	7.11
CLL#110	M	79	0	M	1/1/96	05/23/07				ND	ND	16.92	16.92	5.52	5.52	7.76
CLL#111	M	74	2	M	4/19/05	03/23/10				80	normal 11/2/10	7.63	7.63	2.70	2.70	8.07

Patients were treated with: PCR (pentostatin, cyclophosphamide and rituximab), R-CHOP (rituximab and CHOPS), CAR T cells, R-CVP (rituximab, cyclophosphamide, vincristine, and prednisone), BR (bendamustine and rituximab), LEN (lenalidomine) and (fludarabine, cyclophosphamide, rituximab).

Samples were colected from patients cared for at Memorial Sloan Kettering Cancer Center

M: mutated IGVH gene. **UM:** unmutated IGVH gene

TTFT: Time to first treatment. **OS:** Overall survival
