# Supplementary material for: Musashi 2 influences chronic lymphocytic leukemia cell survival and growth making it a potential therapeutic target
Source: Leukemia. 2021 Jan 27;35(4):1037–52. doi: 10.1038/s41375-020-01115-y (PMC8024198; doi:10.1038/s41375-020-01115-y)
Supplement: Supplementary file 5 — Table S2 [file 41375_2020_1115_MOESM5_ESM.pdf]

Table S2: Antibodies used.

**Anti-Human**	**Fluorochrome**			**Company**
HOXA9	Alexa 488			Abcam (ab191178)
CD19	PE	APC		BioLegend (302208)/BD (340437)
CD5	PerCp Cy5.5	PeCy7		BioLegend (100624, 300622)
CXCR4	PerCp Cy5.5	PeCy7		BiLegend (306516, 306514)
MSI2	Alexa 488			Abcam (ab199067)
Ki-67	PeCy7			BioLegend (652426)
survivine	PE			Cell Signaling (5875S)
Cleaved caspase 3	PE			Cell Signaling (9978S)
p27 Kip1	PE			Cell Signaling (12184S)
p21 Cip1	PE			Cell Singaling (88655)
pp53	Alexa 647			Cell Signaling (8695S)
pAKT	Alexa 488			Cell Signaling (43506S)
pERK	Alexa 488			BD Bioscience (612592)
pBTK	Alexa 647			BD Phosflow (558134)
**Anti-mouse**				
CD45	PerCp			Miltenyi Biotec (130-102 469)
B220	PE			BD Bioscience (553090)
CD5	APC			Miltenyi Biotec (130-106 204)
CD34	FITC			BD Bioscience (553733)
c-Kit	APC Cy7			BioLegend (31322)
