# Supplementary material for: Musashi 2 influences chronic lymphocytic leukemia cell survival and growth making it a potential therapeutic target
Source: Leukemia. 2021 Jan 27;35(4):1037–52. doi: 10.1038/s41375-020-01115-y (PMC8024198; doi:10.1038/s41375-020-01115-y)
Supplement: Supplementary file 6 — Table S3 [file 41375_2020_1115_MOESM6_ESM.pdf]

Table S3 . Mantel Cox regression analyses of TTFT and OS for CLL patients.

A . Univariate Cox regression analyses of TTFT and OS for CLL patients.

	TTFT		OS	
**Prognostic variables**	**Chi-square**	***P*-value**	**Chi-square**	**p-value**
Rai stage	91.363	*0.000	18.681	*0.001
CD38 (positive >30%)	12.209	*0.000	3.860	*0.049
IGHV mutational status	17.999	*0.000	5.347	*0.021
MSI2	15.893	*0.000	19.379	*0.000

B . Multivariate Cox regression analyses of TTFT and OS for CLL patients.

	TTFT		OS	
**Prognostic variables**	**HR (95% CI)**	***P*-value**	**HR (95% CI)**	**p-value**
Rai stage	2.257 (1.669-3.053)	*0.000	2.076 (1.130-3.815)	*0.019
CD38 (positive >30%)	2.111 (1.010-4.412)	*0.047	1.214 (0.284-5.182)	0.793
IGHV mutational status	1.268 (0.567-2.833)	0.564	4.692 (0.923-23.855)	0.062
MSI2	1.869 (0.755-4.630)	0.176	5 (1.076-23. 256)	*0.040

*P > 0.05; TTFT, Time to first treatment; OS, Overall survival; HR, Hazard ratio; CI, Confidence interval.
